# Characterization of pain-related behaviors in a rat model of acute-to-chronic low back pain: single vs. multi-level disc injury

**DOI:** 10.3389/fpain.2024.1394017

**Published:** 2024-05-06

**Authors:** Mary F. Barbe, Frank Liu Chen, Regina H. Loomis, Michele Y. Harris, Brandon M. Kim, Kevin Xie, Brendan A. Hilliard, Elizabeth R. McGonagle, Taylor D. Bailey, Ryan P. Gares, Megan Van Der Bas, Betsy A. Kalicharan, Lewis Holt-Bright, Laura S. Stone, Paul W. Hodges, David M. Klyne

**Affiliations:** ^1^ Aging + Cardiovascular Discovery Center, Lewis Katz School of Medicine, Temple University, Philadelphia, PA, United States; ^2^ Medical Doctor Program, Lewis Katz School of Medicine, Temple University, Philadelphia, PA, United States; ^3^ Department of Anesthesiology, University of Minnesota, Minneapolis, MN, United States; ^4^ NHMRC Centre of Clinical Research Excellence in Spinal Pain, Injury and Health, School of Health and Rehabilitation Sciences, The University of Queensland, Brisbane, QLD, Australia

**Keywords:** low back pain, acute to chronic, disc puncture, algometer, von frey, social interaction skills, open field, pain

## Abstract

**Introduction:**

Low back pain is the most common type of chronic pain. We examined pain-related behaviors across 18 weeks in rats that received injury to one or two lumbar intervertebral discs (IVD) to determine if multi-level disc injuries enhance/prolong pain.

**Methods:**

Twenty-three Sprague-Dawley adult female rats were used: 8 received disc puncture (DP) of one lumbar IVD (L5/6, DP-1); 8 received DP of two lumbar IVDs (L4/5 & L5/6, DP-2); 8 underwent sham surgery.

**Results:**

DP-2 rats showed local (low back) sensitivity to pressure at 6- and 12-weeks post-injury, and remote sensitivity to pressure (upper thighs) at 12- and 18-weeks and touch (hind paws) at 6, 12 and 18-weeks. DP-1 rats showed local and remote pressure sensitivity at 12-weeks only (and no tactile sensitivity), relative to Sham DP rats. Both DP groups showed reduced distance traveled during gait testing over multiple weeks, compared to pre-injury; only DP-2 rats showed reduced distance relative to Sham DP rats at 12-weeks. DP-2 rats displayed reduced positive interactions with a novel adult female rat at 3-weeks and hesitation and freezing during gait assays from 6-weeks onwards. At study end (18-weeks), radiological and histological analyses revealed reduced disc height and degeneration of punctured IVDs. Serum BDNF and TNFα levels were higher at 18-weeks in DP-2 rats, relative to Sham DP rats, and levels correlated positively with remote sensitivity in hind paws (tactile) and thighs (pressure).

**Discussion:**

Thus, multi-level disc injuries resulted in earlier, prolonged and greater discomfort locally and remotely, than single-level disc injury. BDNF and TNFα may have contributing roles.

## 1 Introduction

The World Health Organization defines low back pain (LBP) as pain between the lower edge of the ribs and the buttock that can radiate into other areas of the body, especially the legs (1). LBP is the most common chronic pain condition, is the leading cause of disability globally, and is increasing in prevalence more rapidly than any other chronic pain condition (2–4). This burden and the prevalence and incidence of LBP is higher in women than men (2, 4, 5). Although injury or accumulated damage to the discovertebral complex, annulus fibrosis, internal intervertebral disc structures, facet joints and/or sacroiliac joint can cause acute episodes, the development and maintenance of persistent LBP generally involves complex mechanisms including interactions between the immune and nervous systems. How these mechanisms evolve and relate to pain over time from the onset of acute injury is unclear (6–9).

Animal models have been developed to study mechanisms underlying LBP (see reviews :^10^–^13^). The most common and repeatable method for achieving intervertebral disc (IVD) degeneration is a physical injury of the annulus fibrosis using needle puncture (10, 14, 15). Scraping or surgical blade incision of the annulus fibrosis, injections of inflammatory mediators, DRG or facet joint compression, or applications of various substances (e.g., nucleus pulposus fragments) into discs have also been used to induce IVD injury and/or related pain symptoms (16–24). Partial penetration of the annulus fibrosis induces a slower degenerative process (reduced IVD height and slowly increasing degeneration) than a full penetration (rapid nuclear avulsion but no degeneration) (10, 13), with the former more closely reproducing the human condition of progressive pathology (14). Other studies have focused on the optimal needle size needed for the puncture of rodent discs (25–27). In rats, an 18-gauge needle was optimal for inducing lumbar IVD degeneration and a behavioral index of pain (mechanical hypersensitivity) (25, 27). In contrast, 26-gauge needles failed to cause IVD degeneration, 21-gauge needles produced moderate IVD degeneration but no mechanical hypersensitivity, whereas 16-gauge needles induced acute disc injury but no degeneration. The degenerative effects of single and multiple disc punctures at the same spinal level on histopathology have also been studied, separately, with multiple disc punctures per IVD inducing more symptoms or degeneration than one disc puncture. Only one study in rats has compared the effects of single- (L4/5) vs. multi-level (L2–5) lumbar disc puncture (28). In that study, MRI analyses at 8 weeks post-injury showed similar degeneration of the injured discs regardless of the number of levels injured. However, the study primarily focused on the intervention effects of a drug rather than characterizing differences between single vs. multi-level injury with respect to disc height, histopathology, and pain-related behaviors, among other features, which could have important clinical implications. In humans, the herniation of two lumbar discs (termed tandem disc herniation) on first presentation has a relative low incidence rate, but is not rare (29). That said, migration of the lumbar disc herniation to an adjacent disc (either caudally or rostrally) is a significant predictor of disc herniation recurrence and occurs in up to 19% of patients (30, 31). In contrast, symptomatic multilevel degenerative disc disease in the lumbar region is common in humans (32–34). Thus, research using relevant animal models to compare degenerative, biobehavioral and clinically relevant changes between single and multi-level disc injuries over time in detail and in a controlled manner is needed.

A critical issue in the animal literature is that few studies have examined pain behaviors in IVD injury models, and robust behaviors representative of chronic pain in humans were often not observed. This might be explained by the short duration of follow-up, typically between 3 and 8 weeks after disc injury. This is less than the 3 month time-point in a mouse model of LBP in which injured discs begin to herniate dorsally and develop signs of radiating pain that persist to 12 months post-injury (35, 36). Other work that investigated the long-term impact of different degrees of injury severity (1 vs. 6 “scrapes” of the annulus fibrosis to induce an artificial annular tear) also demonstrated delayed effects. For example, significant low back hypersensitivity, together with induced greater loss of nucleus pulposus, inflammation, and a hypocellular annulus fibrosis with granulation tissue around the needle track, only began from 10 weeks (lasting until study end at 18 weeks) after the more severe “six scrape” injury (17). Together, these data suggest that the rate and severity of disc degeneration, and the resulting behavioral indices of pain, are dependent on both the extent of structural disruption and length of time post-injury. Further long-term studies are needed to support this hypothesis. It remains to be determined in animal models whether multi-level disc injury induces greater physiological degrees of injury severity or greater behavioral indices of pain than single-level injury, and whether those changes correlate.

Tumor necrosis factor alpha (TNFα) is a potent pro-inflammatory mediator that can induce catabolic tissue changes and alterations in cell phenotypes that promote IVD degeneration (37). High levels of TNFα released after IVD injury can sensitize nociceptors and heighten pain symptoms (24, 38, 39), and its inhibition reduces the development of histopathology and pain behaviors in a rat model of disc herniation (40, 41). The generation of pain associated with IVD damage and degeneration also involves neurotrophins, such as brain-derived neurotrophic factor (BDNF), which shares a direct relationship with TNFα and other cytokines (e.g., IL-1β) at this level (37). Although produced at very low levels in the nucleus pulposus and annulus fibrosis of uninjured/non-degenerative IVDs, BDNF expression by these cells is markedly increased in response to injury and degeneration of IVDs in animals and humans (37, 42–45). These increases are thought to be driven, in part, by increased expression of inflammatory factors such as TNFα within the IVD (42, 43, 46), providing support for the hypothesis that BDNF contributes to nerve ingrowth and pain generation in degenerative and injured IVDs (43, 44, 47). Indeed, numerous reports concerning LBP associated with neural ingrowth report associated high levels of BDNF (48, 49). Moreover, BDNF expressed within and around the IVD, like TNFα, has capacity to increase pain signaling mechanisms via driving neuroplastic changes involved in the development and maintenance of central sensitization (50, 51), which contributes to the progression from acute to chronic pain (52–54). Unsurprisingly, higher systemic levels of BDNF (55–59) and TNFα (60–64) are observed in individuals with various chronic pain types and levels are often correlated with pain intensity. However, the timing of systemic responses with respect to pain is unknown for BDNF, and unclear for TNFα; some data indicate that its expression is transient, some indicate that it is at normal levels in patients with IVD herniation and symptoms of sciatica, and others indicate that its early (acute phase) and sustained (over 9 months) expression is predictive of, and associated with, poor long-term recovery (38, 63, 65, 66). Whether changes in BDNF can be detected systemically after lumbar disc injury, and how levels correlate with TNFα and pain as pain evolves from acute to chronic, are unknown.”.

Thus, this study compared musculoskeletal symptoms long-term (18 weeks) from the early-acute onset of injury between rats that received a disc puncture (using an 18-gauge needle) to either one or two lumbar IVDs (i.e., single level vs. multi-level disc injury). We hypothesized that although degenerative changes in an individual disc may be similar, the presence of degenerative changes in two discs would evolve into earlier, prolonged, wider spread (local and remote to injury site) and more behavioral indices of discomfort compared to one. We also examined systemic levels of TNFα and BDNF, and for possible associations with observed behaviors.

## 2 Methods

### 2.1 Animals

Twenty-three Sprague-Dawley, young adult (at least 80 days of age at onset), female rats were used (Taconic Biosciences, Inc., Rensselaer, NY, USA). All experiments were approved by both the University Institutional Animal Care and Use Committee (IACUC, protocol # 4994) in compliance with NIH guidelines for the humane care and use of laboratory animals, and the U.S. Department of Army Animal Care and Use Review Office (ACURO, protocol # CP190070.e001) in compliance with the Department of Defense Instruction 3216.01 (Use of Animals in DoD Conducted and Supported Research and Training), and the US Army Regulation 40-33 (The Care and Use of Laboratory Animals in DoD Programs). We were vigilant about all health and illness issues that might confound our interpretation and that induce unneeded stress on the animal. Behavioral and physical changes consistent with distress, such as squinting, hunching, head tucking, vocalization, as well as physical changes (e.g., weight loss of ≥15% or suture site healing issues), were tracked daily. Clinical medical issues were brought to the immediate attention of the University Laboratory Animal Resources staff, if observed. Rats were weighed at baseline prior to surgery and every 3 weeks thereafter.

### 2.2 Disc puncture surgery

A mechanical discogenic LBP model was adapted from Muralidharan et al. (67) Eight underwent a mid-sagittal puncture of one lumbar IVD (L5/6, DP-1 rats), and 8 received disc punctures to two adjacent lumbar IVDs (L4/5 and L5/6, DP-2 rats), using a similar ventral approach and needle puncture as previously described for rodents (25, 35, 36). Animals were anesthetized with isoflurane prior to surgery (5% induction and 2.5% maintenance, with O_2_ as a carrier). Meloxicam (1–2 mg/kg body weight) was provided one day prior to and the day of surgery. Pre-emptive topical lidocaine was also provided immediately pre-surgery. Access to the lumbar disc space was made using a midline ventral abdominal incision and then by gently retracting the abdominal viscera. The lumbar L4/L5 and L5/L6 IVDs were punctured once per disc to a depth of 2 mm using an 18G needle (using prior markings on the needle to indicate depth of penetration), in the mid-sagittal plane. This depth (2 mm) was chosen to match a study showing moderate IVD degeneration and behavioral signs of LBP following single disc injury (67), and other rat models of IVD injury (67, 68). After IVD puncture, peritoneal and muscle layers were closed using 4-0 coated vicryl (polyglactin glo) sutures. The skin layer was closed using 4-0 Perma-hand silk 4-0 sutures. Animals were kept warm on a rodent warming heating pad (model 7100-53814, Stoelting, Wood Dale, IL, USA; pre-programmed to 37°C) and monitored closely during post-surgical recovery for at least 3 h. In addition, 8 rats underwent anesthesia, abdominal opening, similar movement of the viscera, and then closure without disc puncture, and were termed Sham DP rats. Rats were checked twice daily for the first week after injury, and daily thereafter. Stitches were removed 10–12 days after surgery. Meloxicam (1–2 mg/kg body weight) was provided for 2–3 days post-surgery, as was topical lidocaine. Animals were rested for 3 weeks following surgery to enable healing.

One of the DP-1 rats developed complications due to anesthesia during surgery and died, reducing the number of DP-1 rats to seven and the total number of study animals to 23.

### 2.3 Behavioral testing

A code system was used to identify injury type so that behavioral testing could be performed with testers naïve to group assignment. This code was maintained until statistical analyses were performed. Animals were acclimated to the room for one week after receipt into the facility. All animals were then acclimated prior to study commencement to the assay apparatuses and experimenters over the course of at least 2 weeks, with at least 30 min of acclimation to each assay apparatus, prior to any data collection.

We performed a battery of behavioral phenotyping tests to assess general health and physical indices of stress, pain-like behaviors at sites local and distant to the site of injury, spontaneous pain-like behaviors, and psychosocial behavioral testing as a means to also collect information on general well-being (69–72). Similar to other groups examining symptoms associated with IVD injury in rodent models, we examined general health by assaying body weight across time (67), evoked hypersensitivity to pressure at the site of injury (25, 29, 36) using a pressure algometer applied to the lower back (67), evoked pain-like behaviors at sites distant to the site of injury using the same pressure algometer but applied to the upper thigh and von Frey monofilament assays on the hindpaws (21, 25, 28, 29, 36, 61, 67, 73), and spontaneous pain-like behaviors such as reduced/impaired locomotion during open field gait assays (29, 51) and grooming changes post-IVD injury (15, 74) as changes in grooming may be indicative of stress and pain in rodents (15, 71, 74). We also examined cold sensitivity as similarly performed by others examining symptoms associated with IVD injury (36). However, we used an apparatus (temperature place preference testing apparatus) that allowed us to assess cold sensitivity as a spontaneous pain-like behavior as done previously in our laboratory in other rodent models of injury and disease (75, 76). We extended beyond these commonly used tests to include observational scoring of spontaneous pain-like behaviors that might occur during any evoked or spontaneous pain test, social behavioral testing with a novel rat as a means to gather information on psychosocial behavioral changes, and the scoring of physical indices of stress, each described in Table 1 and as advised for behavioral phenotyping methods of rodents (69–72), and as used previously in our lab for other pain evoking injuries and disorders in animal models (72, 75–81).

**Table 1:** Scoring of altered and abnormal behaviors, and physical indices of stress.

Measure	Description/Feature	Scoring
Observations in arena or testing chamber		
Transfer arousal (1 min after transfer) into arena or testing chamber	Normal (0) vs. prolonged freeze (>10 s), hyperactivity (vigorous rapid/darting movement), escape behavior, or restraint resistance when transferring rat into arena or chamber	1 point each; scored as sum of behaviors observed
Altered/abnormal spontaneous behaviors	Excessive licking of vaginal area, excessive grooming, rearing, escape behaviors (after first minute), vocalization during testing (or excessive vocalization if during pressure sensitivity testing), or aggression towards tester	1 point each if present; scored as sum of behaviors observed
Social Interaction testing—Positive interactions with novel adult female rat	Grooming, licking, or genito-anal sniffing the novel rat, as well as crawling over or under the novel rat	1 point each if present; scored as sum of behaviors observed
Social Interaction testing—Negative interactions with novel adult female rat	Grooming separately from the novel rat; biting, grabbing, pushing, boxing or mounting the novel rat; having to restrain the experimental rat due to excessive aggression towards the novel rat; vocalization when attempting to interact with the novel rat; or increased defecation or urination	1 point each if present; scored as sum of behaviors observed
Urination or defecation (relative amount) during testing	Normal or increased	Absent (0), present (1)
Motor Abilities		
Gait in arena	Normal (0) vs. hesitation (momentary or brief freeze), prolonged freezing (>10 s), limited movement or a halt of movement	1 point each; scored as sum of behaviors observed
Limb or paw paralysis/lameness	Normal (0) vs. uneven steps, limb lameness (foot drags or missteps), or clenched hindpaw	Absent (0), present (1); description
Open field distance	Total distance covered during gat testing	Quantified from foot prints
Stride Length	Average stride length of right and left hind paws	Quantified from foot prints
General Health—physical index of stress of abnormal		
Body weight	>10% loss	1 point
Fur condition	Matted or discolored (1), hair loss (1)	1 point each

Operators performed the pain-related and psychosocial behavioral testing at baseline and then at 3 weeks, 6 weeks, 12 weeks, and/or 18 weeks, after surgery. No more than 3 tests were performed on each animal on a given day to avoid fatigue or stress. If the tests were considerably lengthy, such as monofilament testing, only one type of testing was performed. If unexpected pain/distress was observed, the animal was returned to its home cage and tested on another day. Thus, animals underwent either 3, 2, 1 or no tests on each day of the experiment.

#### 2.3.1 Mechanical (pressure) sensitivity at local (lower back) and remote (upper thigh) sites

A specialized Rodent Pincher algometer [SMALGO (SMall animal ALGOmeter), Bioseb Instruments, Vitrolles, France] was used to test mechanical pressure sensitivity locally (i.e., in the region of injury) at the lower back (lumbar) region. The area of the back to be tested was shaved and marked with a skin marker prior to testing to allow replicate testing in the same region (approximately L4–6) across weeks (the mark was refreshed as needed across the weeks). The animal was gently restrained (after being previously acclimated to this type of restraint for at least 5 min) before using the algometer to apply gentle pressure to the skin at an increasing force until the animal withdrew or vocalized. Withdrawal at lower pressures is interpreted as mechanical pressure hypersensitivity (82). This same algometer instrument and technique was used to test mechanical pressure sensitivity remotely at the upper thigh region at baseline, 12- and 18-weeks post-surgery. Excessive vocalization that occurred during mechanical pressure testing of the lower back or thigh regions was recorded and reported as described in Section 2.3.7.

#### 2.3.2 Mechanical (tactile) sensitivity of hind paws

Tactile (mechanical cutaneous) sensitivity of the hind paws was tested in a Plexiglas chamber with a metal mesh floor in the quadruped position. As noted in Section 2.3, animals were habituated to all apparati prior to onset of the experiments. Five animals were placed into a five chamber clear acrylic chamber with a metal grid floor and allowed to habituate to this environment for at least 10 min. The number of hind paw withdrawals to 10 probings per monofilament size was quantified after stimulating the volar aspect of the paw with a series of 5 calibrated monofilaments from a touch-test monofilament set (North Coast Medical Inc, CA, USA) ranging from 0.16, 0.4, 1, 4, and 7.8 (also referred to as 0.16, 0.4, 1, 4, and 8 force grams, respectively; and 3.22, 3.16, 4.08, 4.56, and 4.93 evaluator sizes, respectively). Hind paw withdrawals were defined as elevating the hind paw, elevating the hind paw and licking it, and elevating the hind paw and shaking it. Probing was performed only if the paw was in contact with the mesh floor. Hind paws were tested bilaterally. Filaments were applied one paw at a time to the mid-palmar surface of the paw through the mesh floor until the filament bent slightly and was kept in this position for approximately 5 s, beginning each time with the lowest sized filament and then sequentially applying the probe once to the right hind paw of all rats before applying the probe once to the left hind paws of all rats. Each filament test was repeated 10 times per session for each hind paw and the animal was allowed to rest approximately 3 min between each of the 10 trials per filament and per hindlimb. Data from the right and left hind paws were averaged for each week assayed before analyses, and the mean number of hind paw withdrawals to 10 probings are reported, as previously used (83, 84). The occurrence of abnormal transfer responses and altered/abnormal spontaneous behaviors were also recorded during this assay (see Table 1) and reported as described in Section 2.3.7.

#### 2.3.3 Temperature place preference testing (thermal two-plate preference test)

Avoidance of cold temperature was assayed in the final week (week 18) using previously described methods (76). Rats were placed unrestrained in an apparatus with two adjacent plates enclosed in a 330 × 165 × 300 mm Plexiglas chamber (T2CT, Bioseb): a reference plate at 22°C (room temperature) and a test plate that decreased in temperature (20–12°C, 4°C per step, 5 min per step, 35 min total). In addition to the acclimation during the initial baseline habitation weeks, rats were allowed to habituate to the chamber for 3 min with each plate at room temperature, prior to starting the temperature changes. The rat was then free to choose their preferred position when moving in the chamber. Movement of the rat across or on the two temperature plates was recorded with an overhead mounted camera connected to a computerized tracking system. This system tallied the time spent on the variable plate, the room temperature plate, and total time in the chamber. Abnormal transfer responses and altered/abnormal spontaneous behaviors were also noted on occurrence during this assay (see Table 1) and reported as described in Section 2.3.7.

#### 2.3.4 Open field gait testing

Rats were allowed to acclimate to this chamber during the baseline habituation weeks. Rats were placed into a clean open field testing chamber for gait testing using footprint pathway methods (the hind paws were inked with different colors). Rats were trained to walk along a 90-cm long, 15-cm wide, paper-covered runway, using previously described methods (72). Total distance travelled during gait testing and the average stride length (measured from all right and left hind paw steps and averaged) were quantified from the inked footprint patterns. Gait in the arena (hesitation, prolonged freezing for >10 sec), limited movement or a halt of movement), and limb or paw lameness (limited movement, limb lameness, or clenched hind paw) were scored as described in Table 1.

In addition, abnormal transfer responses and altered/abnormal spontaneous behaviors during gait testing were noted on occurrence during this assay and scored using adaptations from previously described methods (70, 71, 85, 86) and as defined in Table 1. Behavior scores for gait in the arena, limb or paw paralysis/lameness, abnormal transfer responses, and altered/abnormal spontaneous behaviors were summated and reported as described below.

#### 2.3.5 Social interaction testing

The social interaction test measures sociability and anxiety-like behaviors by assessing how an unfamiliar pair of rats interact in an active social environment (79). Here, this test was performed by exposing the experimental rat to a novel female adult rat during a 10 min observation period. This occurred in a clean and empty rat cage to which each animal was allocated to during the pre-baseline habituation weeks, and to which the experimental animal is allowed to acclimate to for 15 min before introduction the novel female adult rat. On introduction of the novel rat, behaviors are scored on incidence on a scoring sheet. Behaviors classified as positive interactions with a novel rat included: grooming, licking, and genito-anal sniffing the novel rat, as well as crawling over or under the novel rat (see Table 1). Behaviors classified as negative interactions included: grooming separately from the novel rat; biting, grabbing, pushing, boxing, or mounting the novel rat; having to restrain the experimental rat due to excessive aggression towards the novel rat; or increased defecation or urination during the test (see Table 1). The number of positive and negative interaction behaviors were summated separately.

#### 2.3.6 Summated score of altered and abnormal behaviors, and physical signs of stress

Altered, abnormal and negative behaviors observed during any of the behavioral tests were summated. Scored behaviors, if observed, included: excessive vocalization occurring during mechanical pressure testing of the lower back and/or thigh regions; abnormal transfer responses and altered/abnormal spontaneous behaviors occurring during sensitivity testing of hind paws, thermal place preference testing, and/or gait testing; and negative behaviors occurring during social interaction testing. In addition, any observed physical indices of stress (excessive weight loss of ≥10%), the development of a scruffy/dull coat, hair loss, or presence of porphyrin on their heads or limbs) were included in this summated score. These were each scored during each testing event, as described in Table 1, and summated.

### 2.4 Tissue collection, vertebra assessment by x-ray and histology

At study end (18 weeks post-surgery, after final behavioral testing), rats were anesthetized with 5% isoflurane, as described above (Section 2.2), immediately prior to euthanasia. The deeply anesthetized animals underwent thoracotomy and removal of blood by cardiac puncture. Blood was collected into uncoated tubes, allowed to clot for ∼45 min, and then centrifuged at 12,000 revolutions per minute at 4°C for 20 min. Serum (the supernatant) was collected and immediately aliquoted into 200 *μ*l microcentrifuge tubes and stored at −80°C until assayed. Animals were then perfused transcardially with sterile saline first, and then 4% buffered paraformaldehyde. The lumbar vertebra region was collected and postfixed in the 4% buffered paraformaldehyde for 72 h.

A length of vertebra spanning L3-S2 were first assayed using *ex vivo* x-ray imaging methods (Skyscan 1,172 micro-CT instrument, Microphotonics, Allentown, PA, USA). Vertebrae were imaged at 5 µm voxel resolution using a 0.5 cm aluminum filter. The length of vertebra was wrapped in parafilm and mounted in a low-density plastic tube. Multiple images of vertebral segments were stitched together using NRecon reconstruction software (version 2.0, Skycan) into one full image of the vertebra. From these images, an individual IVD height index was determined for both the L4/5 and L5/6 IVDs using DataViewer software (Skycan). IVD height index assays were assessed via x-ray of the vertebral columns prior to decalcification and cryosectioning, using previously described methods (36, 87). In brief, three height measurements were made per IVD and then averaged, before being normalized to the mean heights of the adjacent cranial and caudal vertebral bodies (three measurements per vertebral body). For example, the L4/5 IVD height was normalized to the mean heights of the L4 and L5 vertebral bodies, and the L5/6 IVD height was normalized to the mean heights of the L5 and L6 vertebral bodies.

The vertebrae were then decalcified in RapidCal Immuno (6,089, Statlab.com, McKinney, TX, USA) with every other day changes of this decalcification solution for 3–4 weeks. The vertebrae were then placed in 10% sucrose in phosphate buffer for two days, followed by 20% sucrose in phosphate buffer for two days, before being embedded in OCT compound (4584, Scigen for Fisher HealthCare, Houston, TX, USA). The vertebrae were sectioned longitudinally into 15 micron sections and placed onto coated and charged slides (12-550-15, FisherBrand Superfrost Plus, Fisher Scientific, Pittsburgh, PA, USA). Sections on slides were stored at −20°C until stained with either hematoxylin and eosin (H&E) or Safranin O/Fast Green. The IVDs were then scored using a standardized histopathology scoring system developed by the Orthopaedic Research Society section initiative (13). Five IVD regions and 8 total features were scored according to this system: (1) nucleus pulposus morphology (with shape and area subscores), (2) nucleus pulposus cellularity (with cellularity and morphology subscores); (3) nucleus pulposus-annulus fibrosis border (border appearance); (4) annulus fibrosis (with lamellar organization and tears/fissures/disruptions subscores); (5) endplate (with disruptions/microfracture and osteophytes/ossification subscores). Each feature is scored on a three point scale of 0 (normal) to 2 (most degenerated), according to this system (13). The overall score range is 0–16, with 0 identified as non-degenerated, 3–4 as mild, 7–8 as moderate, and 16 as severely degenerated. Both combined and individual scores for each feature are reported.

### 2.5 ELISA

The serum, collected at 18 weeks post-injury at euthanasia as described above (Section 2.4), was assayed using separate commercially available enzyme-linked immunosorbent assays (ELISAs) for: BDNF (DBNT00, Rat Quantikine ELISA Total BDNF, analytical sensitivity of 1.35 pg/ml, R&D Systems, Minneapolis, MN, USA) and TNFα (EA100366, OriGene, Rockville, MD, USA, analytical sensitivity of <1 pg/ml). Absorbance was measured according to the manufacturer's directions using the Paradigm (Beckman Coulter, Inc., CA) microplate reader. Values below the sensitivity of the test were allocated a score of zero. Data are presented as pg/ml serum.

### 2.6 Statistics

Prior to study onset, a power analysis was performed by a statistician using past nerve injury data generated by MB. We selected representative variables to determine adequate sample sizes: TNFα levels in serum, monofilament tested mechanical sensitivity of hind paws, and temperature place preference data. We chose the most conservative sample size needed to detect differences with an alpha level of 0.05% and 80% power. From this, it was determined that at least *n* = 7/group was needed. A review of the literature on lumbar effects of IVD damage in rodent models report group sizes ranging from 2–12, with an average of 8.3 animals per group (Table 2). Therefore, we strived for *n* = 8/group. Yet, since the original sample size calculation was based on a nerve injury model, which ultimately produced more robust hypersensitivity than the disc injury, the power of our study to detect the observed differences in von Frey was 0.7, which falls short of the recommended 0.8.

**Table 2:** Comparison of our model design and results with previously reported rodent models of lumbar intervertebral disc injury. Table modified and updated from Muralidharan et al, 2017. (67) Sorted by incidence of tactile hypersensitivity, then sex.

Sex/species	Model Induction	Number of animals per group (gp)	Study duration	Body weight changes	Tactile Hypersensitivity	Cold Sensitivity	Pressure Sensitivity	Heat Sensitivity	Gait or Rotarod changes	Changes in other spontaneous behaviors	Ref
Our study											
Female/SD rat	Anterior puncture of either L4/5 (DP-1) or L4/5 and L5/6 (DP-2) using a 18G needle penetrating 2 mm deep	*n* = 7–8/gp	18 wks	Yes	Yes	Yes	Lumbar region and upper thigh: Yes	NA	Yes: Gait NA: Rotarod	Yes (decrease in positive social interaction behaviors, and altered behaviors during open field gait testing)	-
Previous rodent models of IVD injury											
Female/SD rat	Anterior puncture of L4/5 and L5/6 IVDs, using a 18G, 21G or 23G needle penetrating 2 mm deep per group	*n* = 10–12/gp per needle gauge	8 wks	NR or NA	Yes	NA	Yes: Hind paw	NA	NA: Gait Yes: Rotarod	NA	[Glaeser et al. (25)]
Female/SD rat	Posterior puncture of L5/6 IVD using a 0.5 mm diameter needle penetrating 3 mm deep, plus sweeping the needle in the nucleus pulposus 1 to 6 times	*n* = 10–12/gp	18 wks	NR or NA	Yes	NA	Yes: Lumbar region	NA	No Additional: significant declines in forearm grip strength	No	[Lillyman et al. (17)]
Male and Female/SD rat	Annular puncture of L3/4, L4/5, and L5/6 using a 26G needle with injection of TNFalpha (depth not reported)	*n* = 12/gp	6 wks	NR or NA	Yes: Males No: Females	NA	NA	NA	NA	NA	[Mosley et al. (21)]
Male/SD rat	Posterior or Anterior annular puncture of L4/5 IVD using a 21G needle penetrating 3 mm deep	*n* = 10/gp	56 days	NR or NA	Yes: Posterior No: Anterior	NA	NA	Yes, but minor: Posterior & Anterior	No	NA	(Li, Yang, et al. (14); Li, Liu, et al. (39))
Male/SD rat	Annular puncture of L3/4, L4/5, and L5/6 IVDs, 26G, at either a depth of either 1.5 (shallow) or 3 mm (deep) plus injection of PBS, TNFα, or NGF/VEGF	*n* = 6/gp	42 days (6 wks)	NR or NA	Yes, for both shallow and deep punctures	NA	NA	NA	NA	NA	[Lai et al. (73)]
Male/SD rat	Application of CFA to L5 spinal nerve	*n* = 4–7/gp	14 days (2 wks)	No	Yes	NA	NA	Yes	NA	NA	[Amaya et al. (22)]
Male/SD rat	L5/6 facet joint compression injury	*n* = 6/gp	28 days (4 wks)	NR or NA	Yes	NA	Yes: Lumbar region	NA	NA	NA	[Henry et al. (24)]
Male/SD rat	Either: (1) Removal of L4/5 facet joint and then puncture of L4/5 IVDs or (2) Removal of facet joint and then posterior puncture of L2/3, L3/4, L4/5 IVDs; each using a 21G needle penetrating 3 mm deep. Then administration of PBS or GDF6	*N* = 5/gp for pain testing	5–8 wks	NR or NA	Yes	NA	NA	Yes	NA	NA	[Cui et al. (28)]
Male/SD rat	Midline puncture of L3/4, L4/5, and L5/6 IVDs using a 26G needle penetrating 3 mm deep, and injection of 2.5 ul of PBS	*n* = 6/gp	42 days (6 wks)	No: Non significant reduction in LBP group	Yes	NA	NA	Yes	Yes: Gait	Yes (increased grooming, immobilization and “wet dog shakes”)	[Lai et al. (88)]
Male/SD rat	Lumbar endplate microfracture	*N* = 6–7/gp	7 wks	NR or NA	Yes	NA	NA	NA	NA Additional: significant declines in forearm grip strength	NA	(Wang et al. 2023)
Female/CD1 mice	Anterior puncture of L4/5 IVD using a 30G needle penetrating 0.7 mm deep (results for only sedentary mice reported here)	*N* = 5–16/gp, with a mean of *n* = 10/gp	0.5, 3, or 12 months	Yes	No	Yes	NA	NA	No: Rotorod: Additional: significant declines in forearm grip strength	Running wheel: Yes	[Millecamps et al. (36)]
Male/SD rat	Annular puncture of L4/5 and L5/6 IVDs using a microsurgical drill with a 0.5 mm or 0.8 mm diameter needle penetrating 2 mm deep, and removal of nucleus pulposus	*n* = 12/gp	7 wks	NR	No	NA	Yes	Lumbar region: Yes	No: Rotarod	NA	[Kim et al. (68)]
Male/SD rat	Annular puncture of L4/5 and L5/6 IVDs 5 times using a 26G needle, 2 mm deep Annular puncture of L4/5 and L5/6 IVDs 10x times using a 26G, 2 mm deep	*n* = 2–9/gp, with a mean of 5/gp	49 days (7 wks) 49 days (7 wks)	No No	No No	NA NA	Yes: Lumbar region Yes: Lumbar region	NA NA	No: Gait No: Gait	NA NA	[Muralidharan et al. (67)]
Male/SPARC-null mice	Inactivation of SPARC IVD protein	*n* = 3–15/gp, with a mean of 9/gp	6, 12, and 78 wks	NR or NA	No	Yes	NA	No	No, then Yes: Rotarod. No difference first 6 months, then declines in SPARC miceat >70 wks of age Additional: significant declines in forearm grip strength	NA	(Millecamps et al. 2012)
Female/CD1 mice	Puncture of L4/5 IVD using a30G needle penetrating into the middle of the nucleus pulposus	*n* = 4–10/gp, with a mean of 7/gp	4 days, or 0.5 3, 6 or 12 months	NR or NA	NA	NA	NA	NA	NA	NA	(Lee et al. 2022)
Female/SD rat	Posterior puncture of L4/5 IVD using a 0.4 mm diameter needle, and removal of nucleus pulposus by air injection; depth of needle not reported	*n* = 10/gp	21 days (3 wks)	NR or NA	NA	NA	NA	NA	NA	Yes (increased grooming and “wet dog shakes”)	[Olmarker (15)]
Male and Female/SD rat	Chronic compression of DRG	NR	42 days (6 wks)	NR or NA	NA	NA	NA	Yes	NA	NA	[Hu and Xing (23)]
SD rat (sex not specified)	Stab incisions into L3/4, L4/5 and L5/6 IVDs, with number 11 blade at a depth of 1.5 mm	*n* = 5–10/gp, with a mean of *n* = 6/gp	28 days (7 wks)	NR or NA	NA	NA	NA	NA	NA: Gait Yes: Rotarod, although 2 injured rats showed only slight or no increase	NA	[Rousseau et al. (16)]
SD rat (sex not specified)	Facet joint removal, then: superficial injury by scratching annulus of L4/5 IVD with a 0.4 mm diameter needle, disc puncture using a 0.4 mm needle, or fat application; depth not reported	*n* = 10/gp	3 wks	NR or NA	NA	NA	NA	NA	NA	NA	[Olmarker (19)]

CFA, complete Freund's adjuvant; LBP, low back pain; NA, not assessed; NR, not reported; SD, sprague dawley.

GraphPad Prism 10 (version 10.0.0, Boston, MA, ISA) for macOS was used for statistical analyses and graphing. Behavioral outcomes that were tracked longitudinally were analyzed using repeated measures mixed effects REML (Restricted Maximum Likely) models using the factors *surgical group* (3 groups: DP1, DP2 and Sham DP rats) and *time-point* (3–5 time-points), with longitudinal results compared to baselines using Dunnett's multiple comparison tests, and compared between groups at the same time-points using Tukey's multiple comparison tests. Individual lumbar IVD height index and histopathology outcomes were assayed similarly using a repeated measures mixed-effects model, using the factors *surgical group* (3 groups) and *intervertebral levels* (2 levels: L4/5 and L5/6). Temperature place preference outcomes were similarly using a repeated measures mixed-effects model, using the factors *group* (3 groups) and *temperature* (two temperatures: 12 and 14°C). Normality tests (Shapiro Wilk tests) were performed for serum ELISA results. These ELISA data did not pass the normality tests and were therefore analyzed using Kruskal-Wallis tests followed by Dunn's multiple comparison tests. Correlations between behavioral data and ELISA/x-ray/histomorphometry outcomes were assessed with Spearman's rank correlation tests. For ease of reading, *post hoc* results are provided in the figures. Main effects and interactive effects for the statistical analyses are reported in Table 3.

**Table 3:** Statistical findings from the mixed-effects models.

Intervertebral Discs			
	Surgical group	Intervertebral level	Interaction of the previous two factors
Individual lumbar IVD height index	*p* = 0.001*	*p* = 0.02*	*p* = 0.28
Histopathology	*p* < 0.0001*	*p* < 0.0001*	*p* < 0.0001*
Behavioral Outcomes			
	Surgical group	Time-Point	Interaction of the previous two factors
Body Weight	*p* = 0.52	*p* < 0.0001*	*p* = 0.68
Pressure testing of lower back	*p* = 0.03*	*p* < 0.0001*	*p* = 0.008*
Pressure testing of upper thigh	*p* = 0.01*	*p* < 0.0001*	*p* = 0.02*
Tactile sensitivity (monofilament tests)	*p* = 0.09	*p* = 0.001*	*p* = 0.002*
Distance traveled during gait testing	*p* = 0.35	*p* < 0.0001*	*p* = 0.01*
Mean stride length during gait testing	*p* = 0.33	*p* = 0.69	*p* = 0.73
Positive interactions during social interaction testing	*p* = 0.71	*p* = 0.006*	*p* = 0.005*
Negative interactions during social interaction testing	*p* = 0.59	*p* = 0.0002*	*p* = 0.33
Altered/abnormal behaviors occurring during open field gait testing	*p* = 0.002*	*p* = 0.06	*p* = 0.18
Altered/abnormal behaviors occurring any behavioral assay	*p* = 0.001*	*p* = 0.24	*p* = 0.24
	Surgical group	Temperature (12 vs. 14°C)	Interaction of the previous two factors
Temperature aversion test	*p* = 0.97	*p* = 0.04*	*p* = 0.82

* Indicates statistical significance.

## 3 Results

### 3.1 General animal health

**Figure 1:**
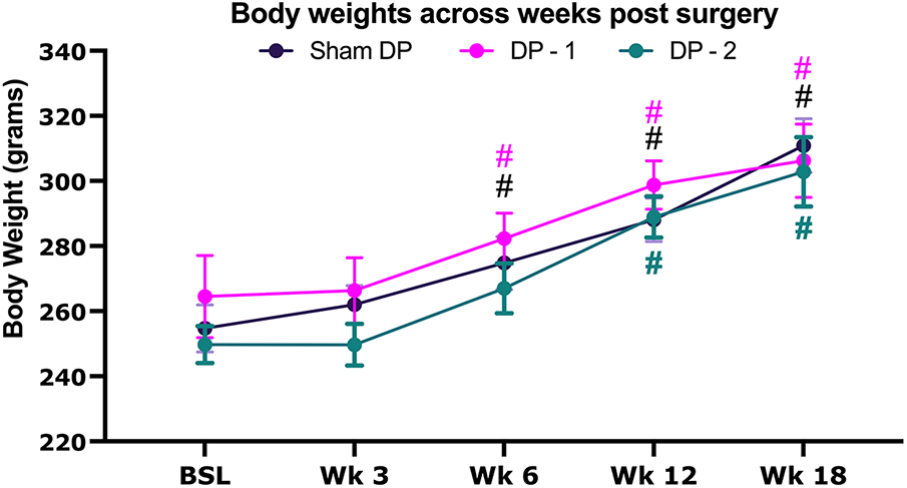
Body weight at baseline before surgery, and then every 3 weeks thereafter for 18 weeks. Sham DP (sham disc puncture control rats), DP-1 [rats that had received puncture injury to only the lumbar (**L**) L5/6 intervertebral disc], DP-2 (rats that had received puncture injuries to both the L4/5 & L5/6 intervertebral discs). #: *p* < 0.05, compared to same group's baseline.

Rats in each group gained weight across the 18 weeks (Figure 1), with statistical differences from baseline observed for sham and DP-1 rats by 6 weeks and DP-2 rats by the 12 week time-point.

### 3.2 Greater loss of lower lumbar disc height in animals with two disc punctures

**Figure 2:**
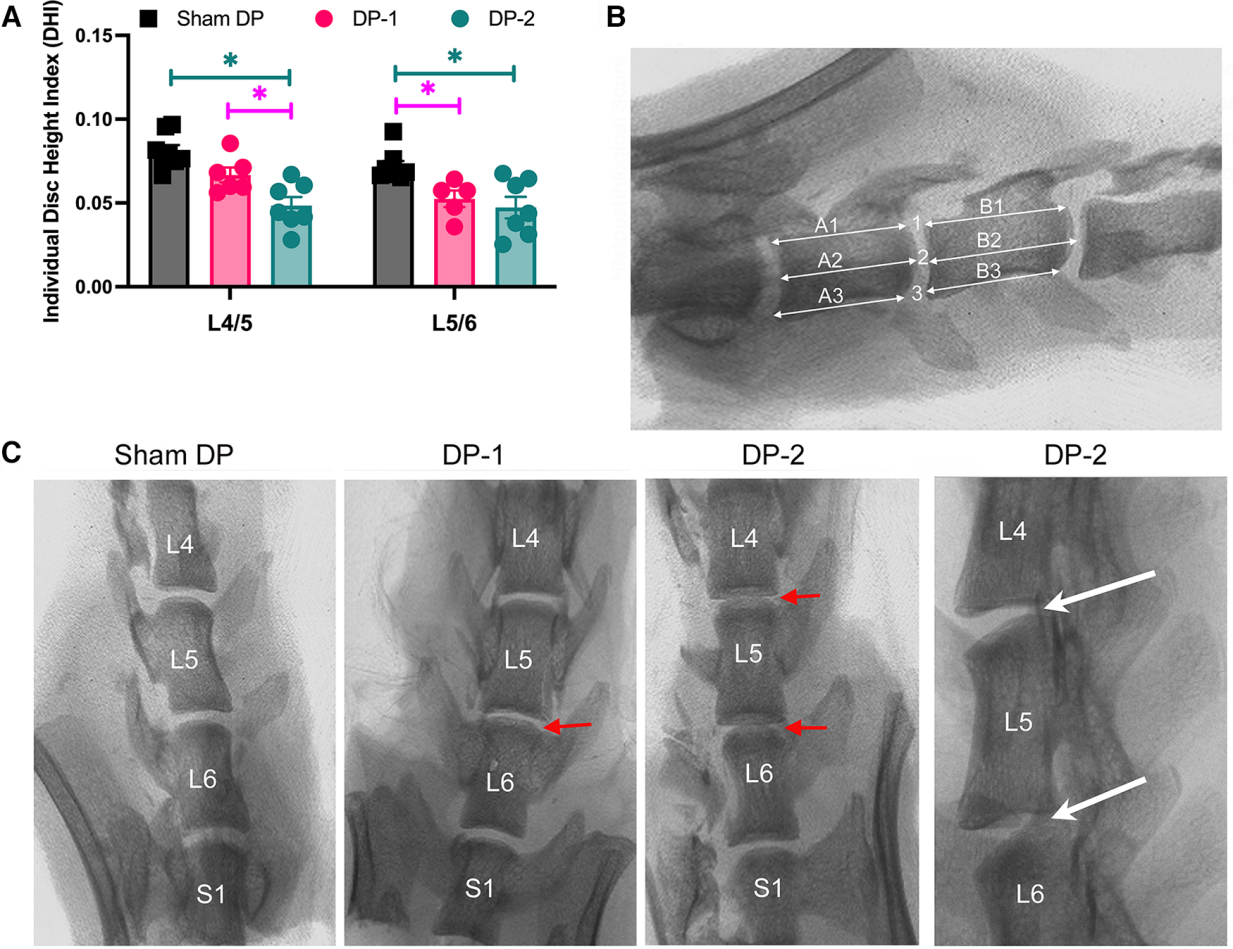
Intervertebral disc (IVD) height measurements from radiographic images. (**A**) Individual disc height index of the IVDs between lumbar vertebrae L4/5 and L5/6. *: *p* < 0.05, compared between groups as indicated. (**B**) Individual disc height index method in which three height measurements were made per IVD and then averaged, before being normalized to the mean heights of the adjacent cranial and caudal vertebral bodies (three measurements per vertebral body) as shown. (**C**) Representative radiographic images of the lower lumbar (**L**) and sacral (S1) vertebrae. The red arrows indicate narrowed discs in DPI-1 and DP-2 rats. The white arrows in far-right panel points out examples of disc wedging. L, lumbar; S, sacral.

Lumbar IVD damage in DP-2 rats was confirmed by x-ray and histologically. DP-2 rats had a lower individual disc height index in L4/5 IVDs, compared to both Sham DP and DP-1 rats (Figure 2A, with measurements taken at sites indicated in Figure 2B and as explained in the methods). Both DP-2 and DP-1 rats had a lower individual disc height index in L5/6 IVDs, compared to Sham DP rats, but no differences between each other. Representative radiological images of lumbar vertebrae and IVDs for each group are shown in Figure 2C. Loss of height in the L5/6 IVD of DP-1 rats and L4/5 and L5/6 IVDs in DP-2 rats are indicated with red arrows in Figure 2C, relative to the greater heights of IVDs in shams DP rats. IVD wedging was occasionally visualized in the injured L4–6 vertebrae of DP-2 rats (Figure 2C, far right panel).

**Figure 3:**
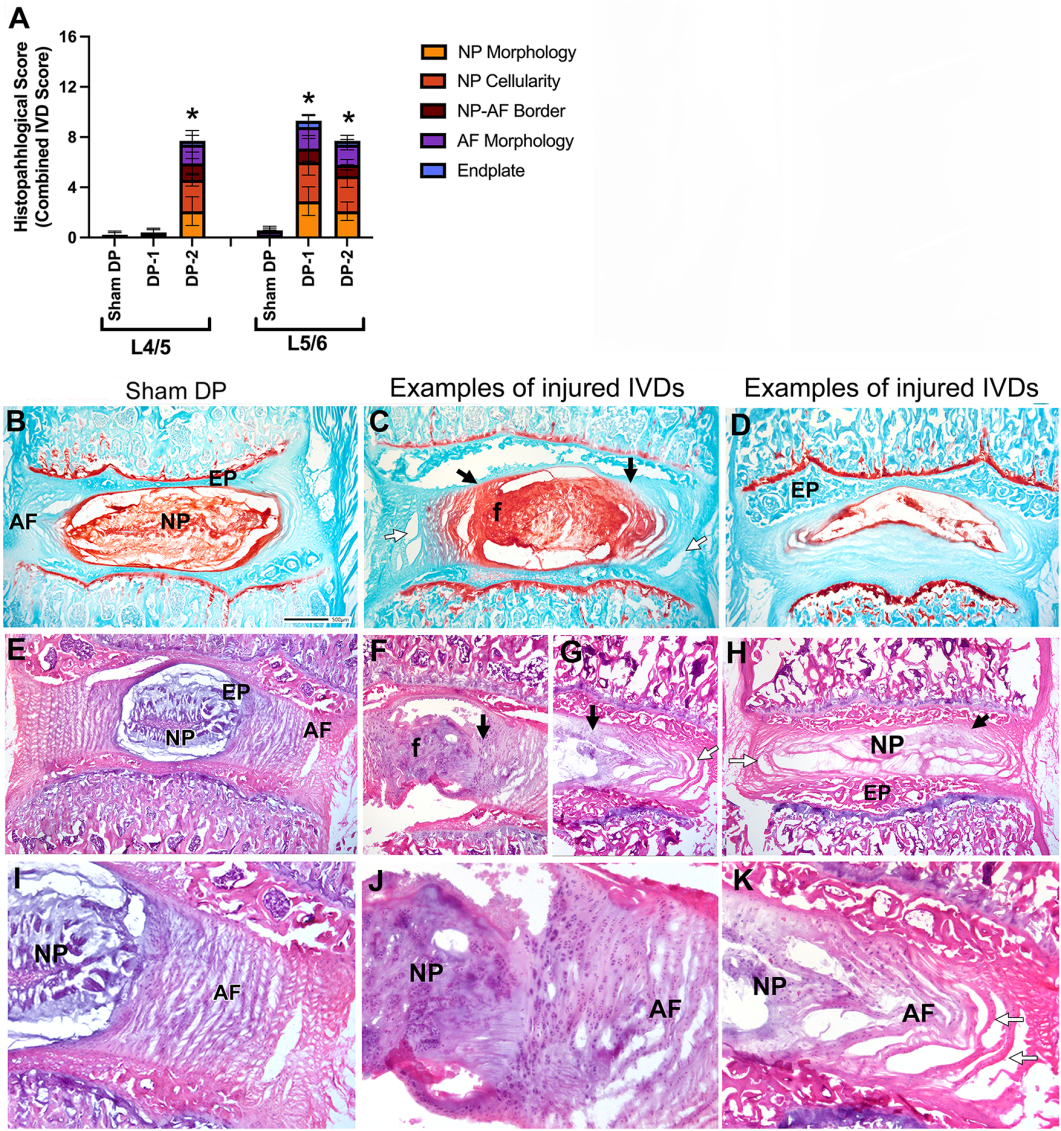
Intervertebral disc (IVD) injury indices: histological assays. (**A**) Combined histopathological IVD scores for each feature assessed in the L4/5 and L5/6 IVDs. (**B–D**) Decalcified and cryosectioned L4/5 and L5/6 IVDs and vertebra stained with safranin O and fast green, with B representative of Sham DP and (**C–D**) representative images of damage seen in injured IVDs. Black arrows indicate regions of merger between the AF and NP, and a loss of their normally separate borders. White arrows indicate areas of disorganization of the AF. The “f” indicates an area of the nucleus pulposus with clear fibrosis. (**E–K**) Decalcified and cryosectioned L4/5 and L5/6 IVDs and vertebra stained with haematoxylin and eosin. Enlarged images of regions from panels (**E–G**) are shown in panels (**I–K**). Panels (**E,I**) are representative images of Sham DP IVDs. Panels (**F,H,J,K**) are representative images of damage seen in injured IVDs (such as fibrosis/granulation of the nucleus pulposus [panels (**F,G,J**)], hypocellularity of the nucleus pulposus [panels (**D,H**)], and disorganization of the annulus fibrosis [panels (**G,H,K**)]. Symbols as above. Asterisks indicate NP regions shown enlarged in the insets. Scale bar in D Sham DP panel is 500 microns and applies to all vertebrae images. AF, annulus fibrosis; EP, endplate; f, fibrosis of nucleus pulposus; NP, nucleus pulposus.

Histological evaluation using Safranin O/Fast green stained sections and H&E stained sections revealed that the punctured IVDs showed signs of moderate degenerative changes, compared to Sham DP rats (Figure 3). Combined IVD histopathology scores were higher for all injured IVDs, compared to un-injured IVDs (Figure 3A). Histologically, sham animals showed typical morphology of their nucleus pulposus region and cells, annulus fibrosis regions with clearly organized lamella, and distinct boundaries between the nucleus pulposus and annulus fibrosis (Figure 3A,B,E,I). The most common degenerative changes in the punctured IVDs of DP-1 and DP-2 rats included fibrosis/granulation of the nucleus pulposus (Figure 3A; indicated with an “f” in Figure 3C,F, and shown enlarged in Figure 3J), rounded and clustered cells in the nucleus pulposus and rounded cells in the nearby annulus fibrosis (Figure 3F,J), a loss of distinct interfaces between the nucleus pulposus and annulus fibrosis (Figure 3A; indicated with black arrows in Figure 3C,F–H, and shown enlarged in Figure 3J,K), and disorganized annulus fibrosis layers (indicated with white arrows in Figure 3G,H and in the enlarged image of Figure 3K). Figure 3D also shows an example of a punctured IVDs with a hypocellular nucleus pulposus region (seen in 2 of 23 total injured IVDs examined in DP-1 and DP-2 rats). No differences were observed between the injured L5/6 IVDs of DP-1 and DP-2 rats, and no degenerative changes were observed in L4/5 IVDs of DP-1 rats (Figure 3A). Main effects and interactive effects for the statistical analyses are reported in Table 3. Supplementary Figure S1 shows the scatter plots of these data separately. Disc herniation was not observed in any punctured IVDs.

### 3.3 Pain related behaviors were more pronounced earlier and for longer in animals with multi-level IVD injury

#### 3.3.1 Increased local (back region, site of injury) mechanical pressure sensitivity

**Figure 4:**
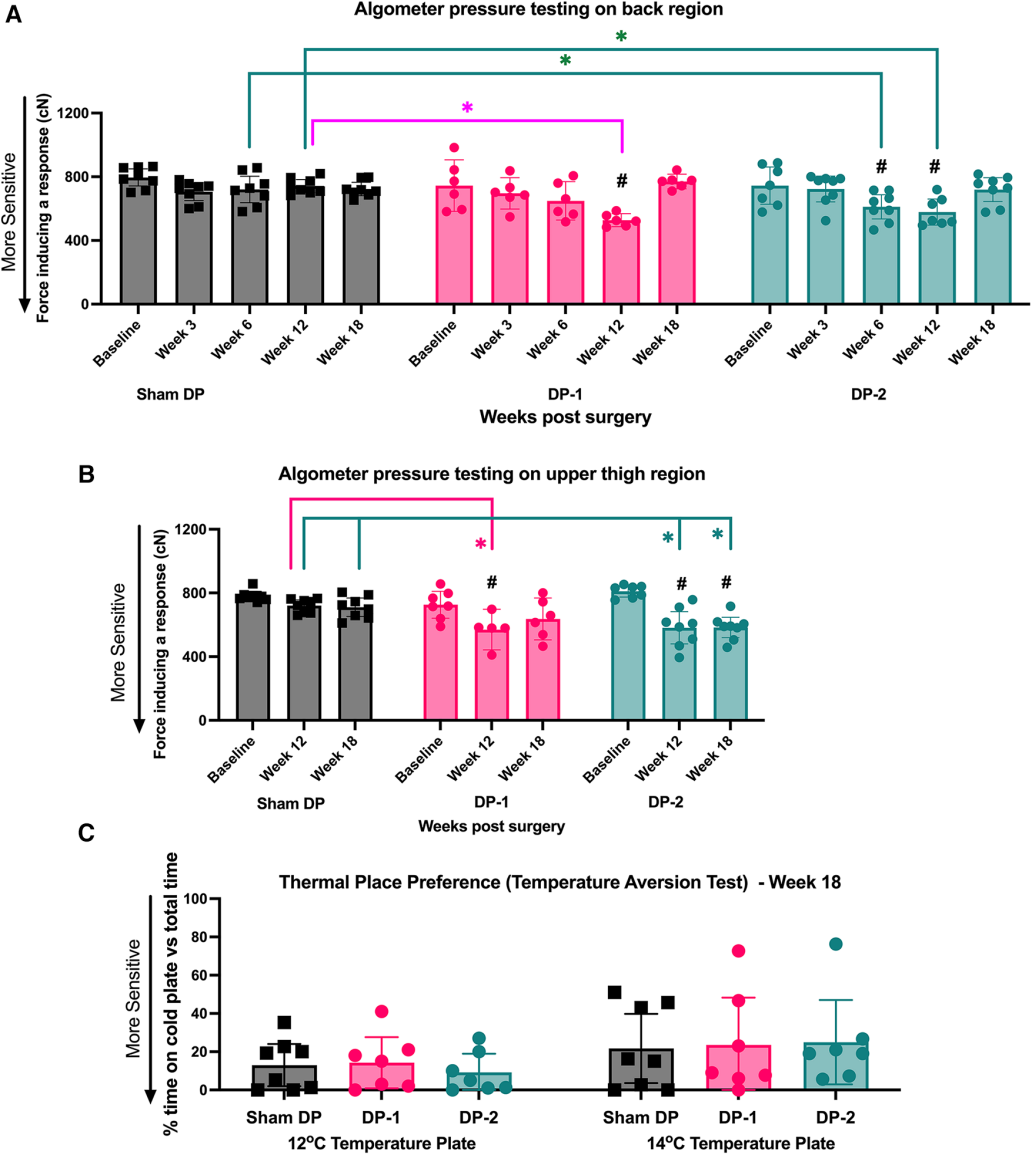
Local (lower back) and remote (upper thighs) pressure sensitivity measured using an algometer, and cold sensitivity measured using a temperature place preference test. (**A**) Algometer test results for the lower back region, assessed at week 0 (baseline), 3, 6, 12 and 18 post-injury. (**B**) Algometer test results for the upper thigh region, assessed at week 0 (baseline), 12 and 18 post-injury. (**C**) Thermal place preference test results, assessed as the percentage of time rats spent on the 14°C and 12°C plates instead of the room temperature plate at 18 weeks (study endpoint). #: *p* < 0.5, compared to same group's baseline; *: *p* < 0.05, compared between groups as indicated.

Local mechanical sensitivity at the lower back was tested using an algometer in which the level of force inducing a withdrawal and/or vocalization response was determined at each testing point. Withdrawal responses and/or vocalization in DP-2 rats were evoked by lower mechanical pressure on their low back region at 6- and 12-weeks post-injury, compared to their baseline and Sham DP (Figure 4A). In DP-1 rats, withdrawal responses and/or vocalization were evoked by lower mechanical pressure on their lower back region at 12-weeks only, compared to their baseline and Sham DP rats (Figure 4A). Values were no different from baseline or between groups at 18 weeks. See also Table 3.

#### 3.3.2 Increased remote (thigh region, remote to site of injury) mechanical pressure sensitivity

Algometer testing of the upper thigh region was performed at baseline, 12- and 18-weeks post-injury. Withdrawal responses and/or vocalization were evoked in both DP groups by lower mechanical pressure on their upper thigh region at 12-weeks post-injury, compared to their baseline and Sham DP rats (Figure 4B). This increased remote pressure sensitivity persisted to 18-weeks post injury in DP-2 rats only (Figure 4B). See also Table 3.

#### 3.3.3 Cold sensitivity was not altered at 18 weeks post-injury

Aversion to noxious cold temperatures was tested at 18-weeks using a two-plate temperature place preference test. Each group, including Sham rats, similarly avoided the variable plate when it reached 14°C or 12°C at 18-weeks (Figure 4C). See also Table 3.

#### 3.3.4 Increased (hind paw) mechanical tactile sensitivity

**Figure 5:**
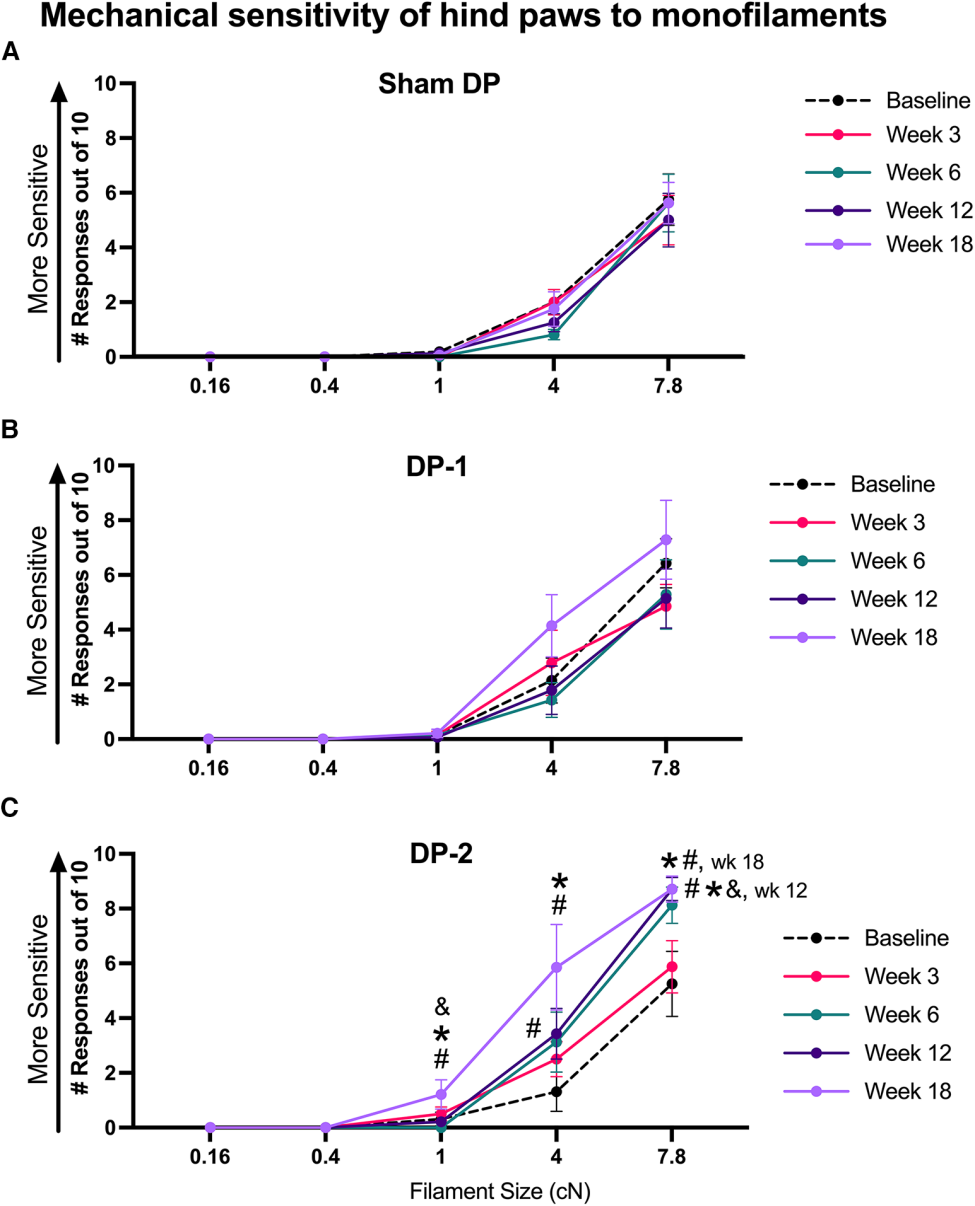
Tactile sensitivity of hind paws reported as number of hindlimb withdrawals to monofilament probing of hind paws (10 probings/filament) at week 0 (baseline), 3, 6, 12 and 18 post-injury to the sizes of monofilaments shown. (**A**) Sham DP. (**B**) DP-1. (**C**) DP-2. #: *p* < 0.5, compared to same group's baseline; *: *p* < 0.05, compared to Sham DP results.

Hind paw mechanical sensitivity was assayed using 5 sizes of monofilaments, and hindlimb withdrawal responses out of 10 probings were counted for each filament size. Sham rats showed similar responses to each filament across the weeks (Figure 5A). DP-1 rats did not show mechanical tactile sensitivity in any week after injury (Figure 5B). In contrast, DP-2 rats responded with a higher number of hindlimb withdrawals in week 12 when tested a 4 cN sized monofilament, compared to their baseline (Figure 5C), and when tested a 7.8 cN sized monofilament, compared to their baseline, Sham and DP-2 rats. In week 18, DP-2 rats responded with a higher number of hindlimb withdrawals when tested with a 1 cN sized monofilament, compared to their baseline, Sham and DP-2 rats; when tested with a 4 cN monofilament, compared to their baseline and Sham DP rats, and when tested with a 7.8 cN monofilament, compared to their baseline and Sham DP rats. See also Supplementary Figure S3 for scatter plots and Table 3.

#### 3.3.5 Decreased distance traveled during gait assays

**Figure 6:**
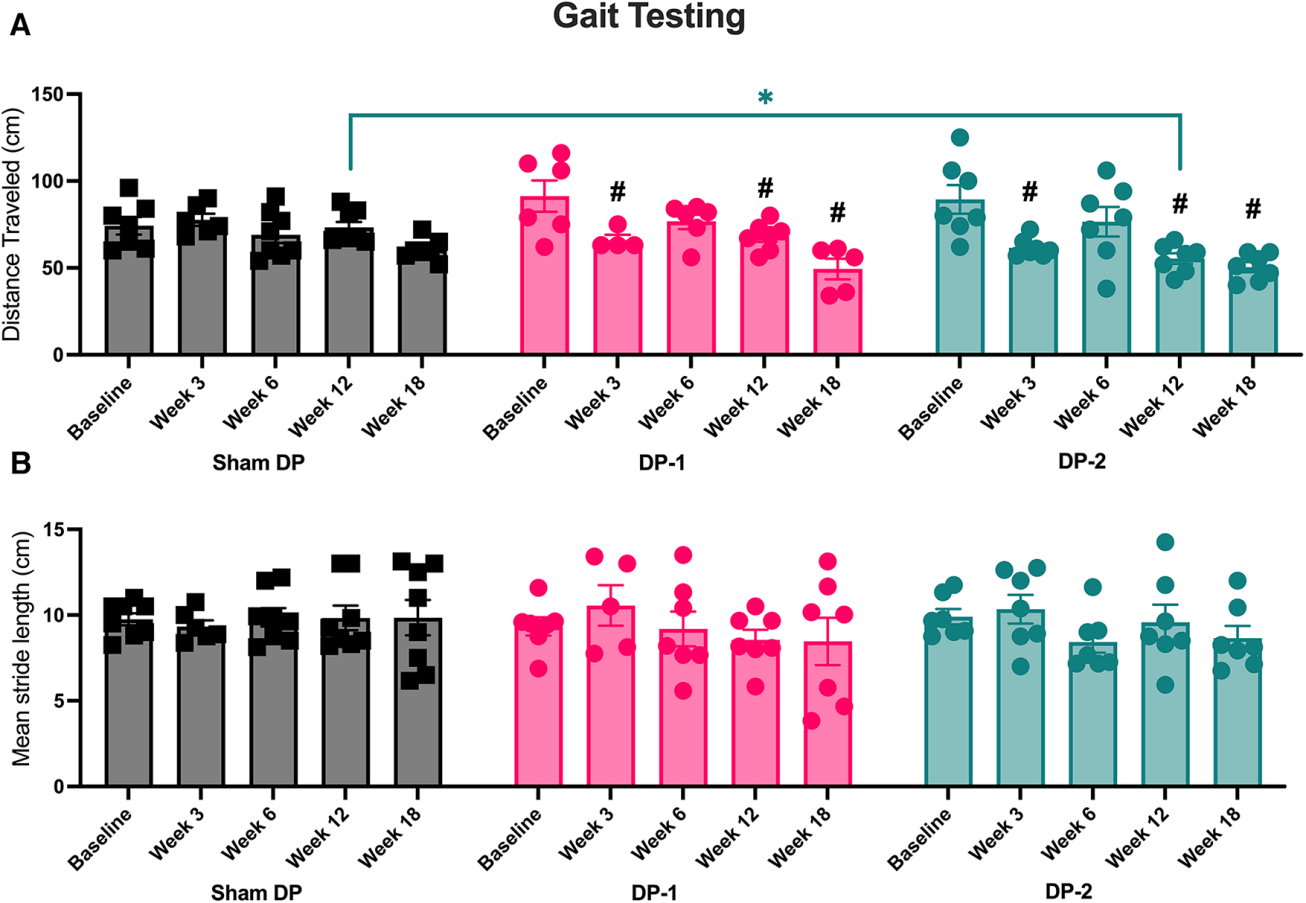
Gait testing using inked footprint methodology at week 0 (baseline), 3, 6, 12 and 18 post-injury. (**A**) Distance travelled at each testing time-point. (**B**) Mean stride length at each testing time-point. #: *p* < 0.5, compared to same group's baseline; *: *p* < 0.05, compared between groups as indicated.

Total distance traveled and stride lengths were assessed with open field gait assays (Figure 6). Both DP-1 and DP-2 rats traveled less distance in weeks 3, 12 and 18 relative to baseline (Figure 6A), and DP-2 rats alone traveled less distance at 12-weeks relative to Sham DP rats (Figure 6A). Mean stride length did not differ between groups or across weeks (Figure 6B). Additional analyses exploring stride length range at each time-point also found no differences between weeks (Supplementary Figure S2). See also Table 3.

### 3.4 Declines in psychosocial behaviors were greatest in animals with multi-level IVD injury, and these were most evident during movement

#### 3.4.1 Positive social behaviors reduced in week 3 in DP-2 rats

**Figure 7:**
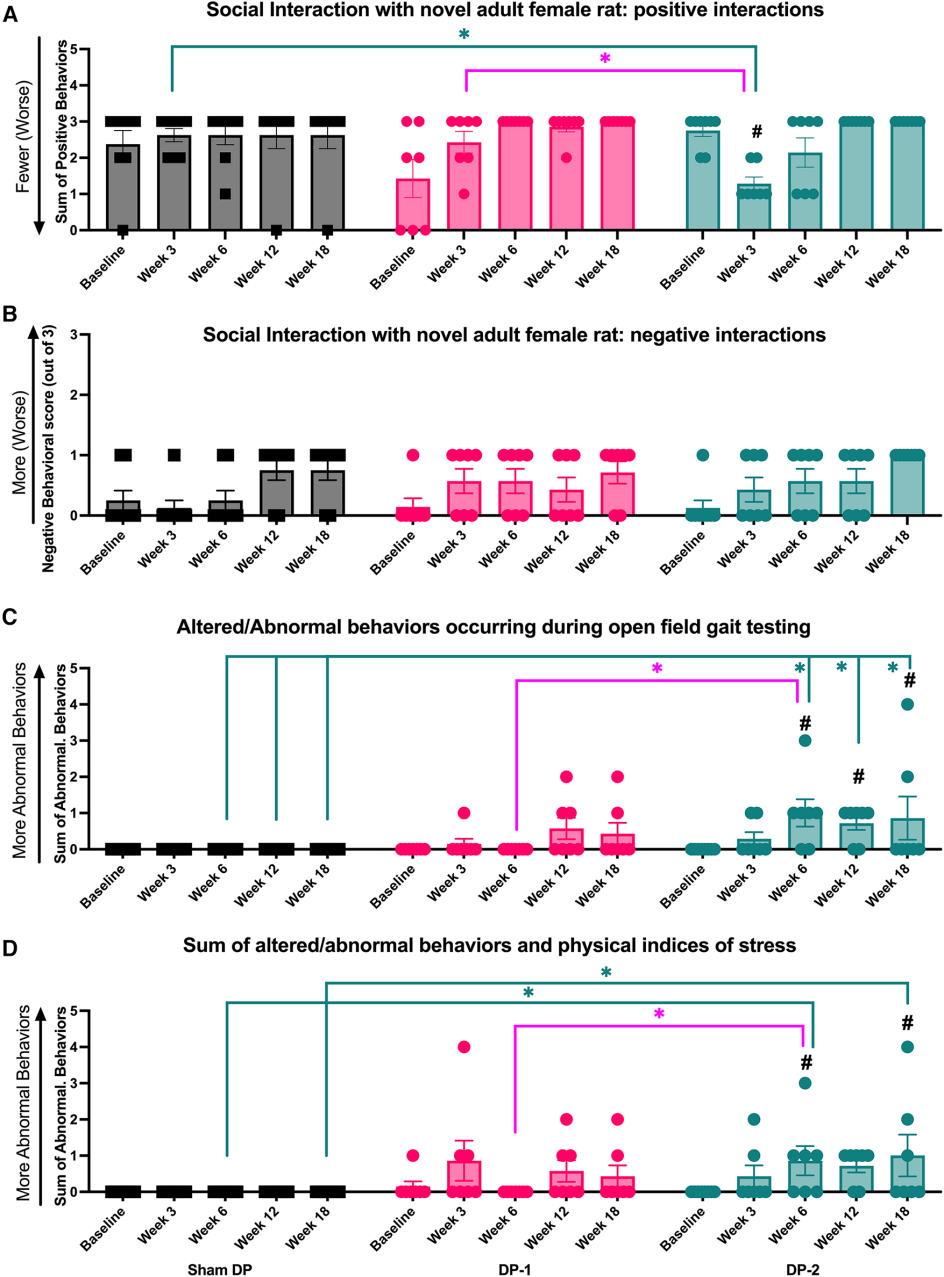
Positive and negative social interaction behaviors, altered and abnormal behaviors during gait testing, and summated altered and abnormal behaviors at week 0 (baseline), 3, 6, 12 and 18 post-injury. (**A**) Positive interaction behaviors during social interaction testing. (**B**) Negative interaction behaviors during social interaction testing. (**C**) Altered or abnormal behaviors during gait testing. (**D**) Summated altered and abnormal behaviors during temperature place preference, gait, and social interaction testing. #: *p* < 0.5, compared to same group's baseline; *: *p* < 0.05, compared between groups as indicated.

Positive interactions between each experimental rat and a novel female rat were on occurrence and scored across a 5 min interval (including, grooming, licking and genito-anal sniffing of a novel adult female rat; see Section 2.3.6). DP-2 rats displayed fewer positive interactions with a novel rat at 3-weeks post-injury, compared to their baseline, Sham DP, and DP-1 rats (Figure 7A).

#### 3.4.2 Negative social behaviors did not differ between groups

Negative interactions with a novel female rat were also noted on occurrence and scored (see Section 2.3.6). No group differences were observed (Figure 7B).

#### 3.4.3 Altered and abnormal behaviors during gait testing increased in DP-2 rats

Altered and/or abnormal behaviors occurring immediately after transfer to the gait testing arena and during gait testing (behaviors described in Table 1) were scored on incidence. DP-2 rats displayed an increase in these behaviors at 6-, 12- and 18-weeks, compared to their baseline and Sham DP rats, and at 6-weeks post-injury, compared to DP-1 rats (Figure 7C). Observed behaviors included: escape behaviors, hesitation before walking, freezing for >10 sec, and refusal to walk.

#### 3.4.4 Summated altered and abnormal behaviors increased in DP-2 rats, and primarily presented during gait testing

The number of altered and/or abnormal behaviors observed during all behavioral testing were summated as were physical signs of stress (described in Table 1). DP-2 rats displayed a greater number of these behaviors and physical signs at 6-, 12- and 18-weeks post-injury, compared to their baseline and Sham DP rats, and at 6-weeks post-injury, compared to DP-1 rats (Figure 7D). Behavioral responses during gait testing accounted for most of these differences (see Figure 7C,D). Other contributing behaviors/factors included: aggression towards a novel rat during social interaction testing in week 3 (one DP-2 rat), escape behaviors during temperature place preference testing in week 18 (one DP-2 rat), and physical indices of stress at 3-week post injury: a 6% weight loss (one DP-2 rat that showed recovery to a similar weight as other rats by week 12) and porphyrin pigmentation on the forehead and nose (one DP-2 rat). Different DP-2 rats displayed these features (i.e., not the same rat per observation).

### 3.5 BDNF and TNFα were elevated at week 18 in rats with multi-level IVD injury, and levels correlated with remote mechanical sensitivity and negative social interaction behaviors

**Figure 8:**
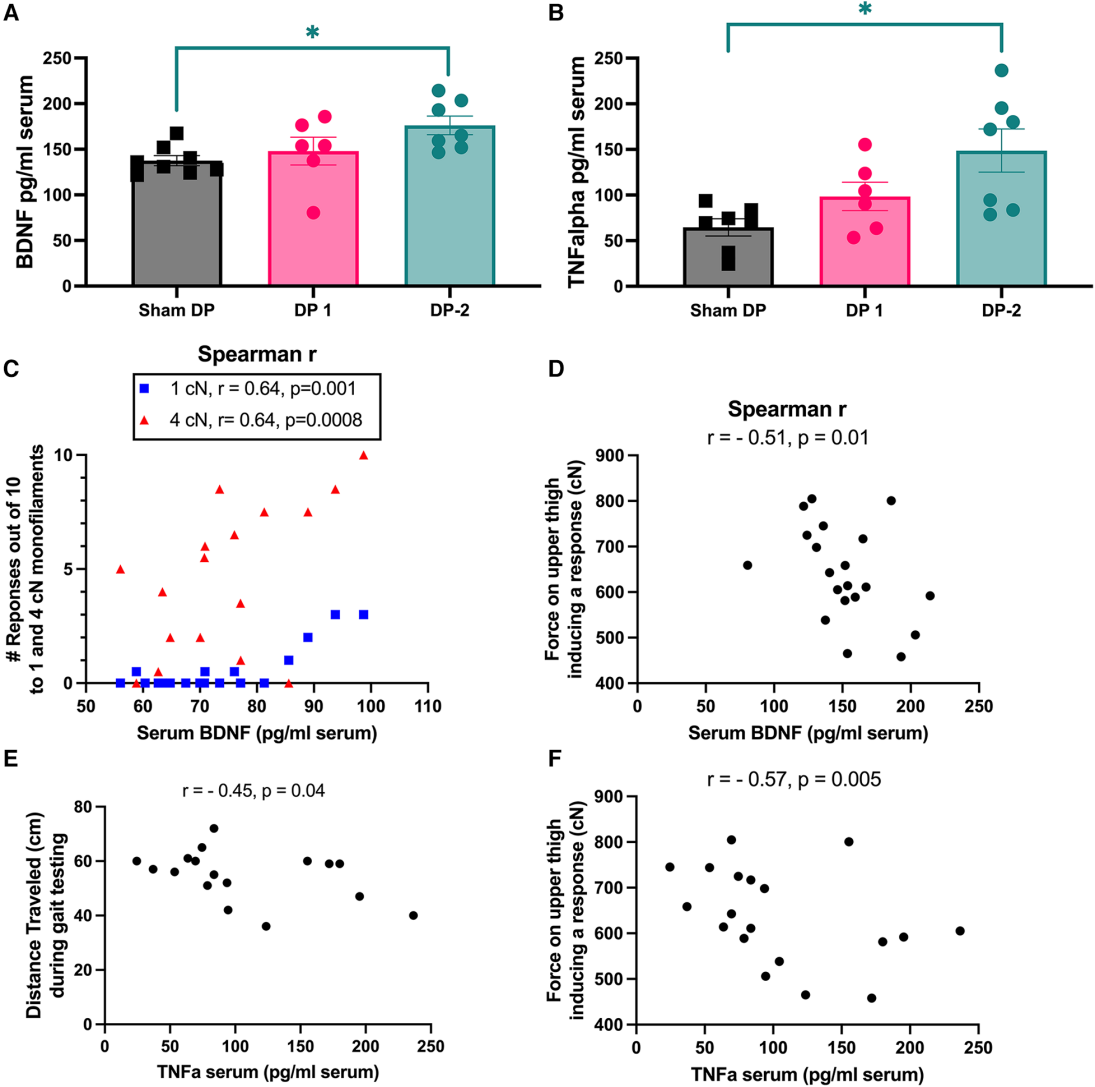
Serum levels of BDNF and TNFα (measured using ELISA) at 18 weeks and significant correlations between these biomarkers and behavioral measures. (**A**) Serum BDNF levels. (**B**) Serum TNFα levels. (**C,D**) Spearman r correlations between serum BDNF levels and hind paw mechanical (tactile) sensitivity to 1 cN and 4 cN monofilaments, and upper thigh mechanical (pressure) sensitivity measured (with an algometer) as the force required to elicit a withdrawal or vocalization response. (**E,F**) Spearman *r* correlations between serum TNFα levels and distance travelled during gait testing, and upper thigh pressure sensitivity, respectively. *: *p* < 0.05, compared between groups as indicated.

Serum levels of BDNF and TNFα were higher at 18-weeks in DP-2 rats, but not DP-1 rats, compared to Sham DP rats (Figures 8A,B). BDNF moderately and positively correlated with the number of hindlimb withdrawal responses to 1 cN and 4 cN monofilaments (i.e., remote tactile sensitivity, *r* = 0.64 each; Figure 8C), and moderately and negatively correlated with the amount of pressure needed to evoke a response on the upper thigh (i.e., remote pressure sensitivity, *r* = −0.51, Figure 8D). TNFα levels correlated moderately and negatively with distance traveled during gait testing (*r* = −0.45. Figure 8E), and with remote pressure sensitivity on the upper thigh region (*r* = −0.57, Figure 8F).

### 3.6 Some measures of IVD degeneration and pain behavior correlated

Some significant correlations between histological indices of disc degeneration and behavioral measures of pain or discomfort were observed. Individual disc height indexes for L5/6 IVDs moderately and positively correlated with remote (upper thighs) pressure sensitivity (such that less pressure was required to evoke a response in animals with lower L4/5 IVD heights, *r* = 0.56). Histopathology scores for the L5/6 IVDs correlated moderately and negatively with remote pressure sensitivity (upper thighs, *r* = −0.61) and distance traveled during gait testing (*r* = −0.63).

## 4 Discussion

Our objective was to determine whether long-term (18 weeks) pain-related behaviors evolved differently in an acute-to-chronic LBP model induced by injury to either one or two lumbar IVDs. As summarized in Table 2, only one prior study has examined the effects of single versus multi-level IVD damage; the length of that study was 5–8 weeks, depending on the sub-experiment (28). In general, behaviors indicative of pain and/or discomfort presented earlier and were more pronounced in rats with a multi-level (DP-2) vs. single level (DP-1) IVD injury. By 3 weeks post-injury (first follow-up time-point), the distance travelled during gait testing had decreased similarly for both DP groups, yet DP-2 rats also showed a reduction in positive social interactions with a novel adult female rat. By 6 weeks, pressure sensitivity local to the site of injury (i.e., lower back), and altered/abnormal behaviors during gait testing presented in DP-2 rats (not DP-1 rats). By 12 weeks, local (lower back) and remote (thigh) pressure sensitivity, and gait distance travelled, had worsened/declined in both DP groups. Also at 12-weeks, DP-2 rats presented with remote tactile sensitivity (hind paws), and continued to present with altered/abnormal behaviors during gait testing (behaviors not observed in DP-1 rats). By 18-weeks, local pressure sensitivity had resolved in both groups. However, in DP-2 rats, remote pressure sensitivity and altered/abnormal behaviors during gait testing persisted, and remote tactile sensitivity (hind paws) was evident to several sizes of monofilaments. The 18-week behavioral changes in DP-2 rats were accompanied by higher circulating levels of BDNF and TNFα, compared to Sham DP rats, and these levels correlated positively with remote pressure and tactile sensitivity, and with less distance traveled during gait testing. Moreover, greater remote pressure sensitivity and less distance traveled during gait testing correlated with radiological and histological indices of disc degeneration. In summary, disc degeneration resulting from the puncture of two IVDs was accompanied by earlier local sensitivity, enhanced and/or prolonged remote sensitivity and several other pain-behavioral symptoms, and systemic neuroinflammatory responses, relative to the puncture of one IVD.

In this study, a single IVD puncture was given to either the L4/5 IVD alone (DP-1 rats) or to both the L4/5 and L5/6 IVDs (DP-2 rats) using an 18G needle, which was inserted to a depth of 2 mm (with no histological evidence of needle penetration through the annulus fibrosis into the nucleus pulposus) without disturbing the spinal roots using a ventral approach. This resulted in a reduction in height in the L5/6, but not L4/5, IVD in DP-1 rats, and both L4/5 and L5/6 IVDs in DP-2 rats. Histologically, the punctured IVDs from both DP groups showed radiological and histological signs of degeneration at 18 weeks post-injury. Similar histopathology was observed in the punctured IVDs for both DP groups, including: fibrosis of the nucleus pulposus [also termed granulation (19)], annulus fibrosis layer disruption and moderate bulging (avoiding complete disc herniation), and merging of the normally separate interface between the nucleus pulposus and annulus fibrosis, each a sign of disc degeneration in rodents (13, 89). It was our intent to perform a moderate IVD injury with a slower progressive degeneration than observed in models using larger needles, deeper insertion of needles, and/or approaches that displace adjacent nerve roots, as these methods can result in complete disc rupture as early as 24 h after surgery (19, 27, 90), which is a far less common clinical scenario. For example, one study examining the effects of puncturing IVDs with either a 16G, 18G or 26G needle in rats showed that the 16G needle induced both degeneration and herniation of the IVDs within 1 week of the procedure (rather than the usually slower onset of degenerative changes observed in humans), whereas the 26G needle induced no histopathological changes (27). The 18G needle inserted to a depth of 2 mm, used in this study, induced a disordered annulus fibrosis, indistinct interfaces, and nucleus pulposus replacement by fibrotic tissues, but not early or major nucleus pulposus herniation. In the event of complete disc herniation, leakage of the nucleus pulposus and its bioactive contents into the spinal canal would occur, which is thought to induce nodules, Schmori's nodes, and osteophyte formation associated with severe IVD injury (19). We suspect that some nucleus pulposus leakage occurred in our model as nucleus pulposus granulation, which occurred here and is not induced by superficial puncturing of the disc (19). Only deeper DP or exposure to the nucleus pulposus contents (via experimental application) stimulates this level of degeneration (19). We observed no degeneration of the adjacent unpunctured L4/5 IVD in DP-1 rats at 18 weeks post-injury. This contrasts with findings by Millecamps and colleagues (36) who examined these structures in mice 12 months after disc puncture. Although species or other model differences may explain some of this difference, we hypothesize that injury-induced mechanical (instability) changes and activation/release of bioactive substances within the nucleus pulposus would more likely negatively impact adjacent IVDs over 12-months than our much shorter 4.5-month timeframe.

Pressure sensitivity at the lower back, as similarly tested in our study (using algometry), has been assessed in a rat model that involved needle puncture of the L5/6 disc followed by one or six sweeps of the needle while within the disc to induce an artificial annulus tear (17). They observed greater local sensitivity to pressure at 10 and 16 weeks post-injury, as well as declines in forelimb grip strength (a sign of discomfort during lower back movements as the test involves a task that evokes back movement) at 6, and 10–18 weeks, but, unlike our observations, no change in total difference traveled during gait testing (Table 2). We do not know if this behavioral difference is explainable by differences in the disc injury model or gait testing. In another study, puncturing of the L4/5 and L5/6 IVDs five or ten times per disc in rats led to the development of local (lower back) noxious pressure hyperalgesia beginning at 14 days post-surgery that persisted to study end at 49 days post-surgery (Table 2) (67). The earlier onset of local hypersensitivity relative to our study (i.e., increased pressure sensitivity at the lower back by week 6 in DP-2 rats) is likely explained by the higher severity of the injury in that study—i.e., one vs. five or ten punctures per IVD. Injury to the lower lumbar discs has also been shown to induce declines in walking abilities (16, 91), again without differences in step length (91), matching our results, as well as increased grooming and wet dog shakes (15), which we did not record (Table 2). Yet surprisingly, despite histological and radiological evidence of IVD degeneration, local mechanical pressure sensitivity had resolved by 18-weeks in our DP rats. This may reflect aspects of local tissue healing and repair, as shown previously in rats whereby some degenerative processes stabilized at 4 weeks (collagen III and Sox9 levels) but others (e.g., collagen I increases) persisted (26).

Increased sensitivity at regions remote to the injury site was also displayed by DP-2 rats, including to pressure of the upper thighs (weeks 12 and 18 post-injury) and tactile probing of the hind paws (weeks 12 and 18 in DP-2 rats). As shown in Table 2, tactile hypersensitivity (also termed mechanical allodynia in the literature) is a common symptom, yet not always present in various rodent models of IVD injury. Some literature suggests that vertebrogenic and discogenic LBP can include the presence of pain patterns into the lower extremity (1, 92), whereas other suggest that it does not involve pain below the level of the knee (93). Many studies in humans with LBP exclude those with symptoms of radicular nerve involvement (93), hindering a full understanding of this diagnosis (6). Disc herniation and even bulging can also lead to enhanced pain symptoms as a consequence of mechanical deformation of the disc on adjacent nerve roots. However, as our model does not directly involve nerve damage and resulting radiating symptoms, we and a growing body of work point to changes within the central nervous system as a potential driver of these distal sensory changes. These changes are proposed to enhance neuronal responsiveness in central pain pathways, a process referred to as central sensitization (54). Although direct evidence of central sensitization in humans is difficult to measure, many studies report enhanced/altered sensory responses in regions unrelated to the painful/injured area in people with chronic LBP without nerve damage, including the foot (94), forearm and thumb (53). These measurable signs that may be attributed to central sensitization have been identified as early as the acute stage (<2 weeks of onset) of LBP (53, 95), countering the traditional clinical belief that central sensitization develops much later in the transition from acute to chronic pain. Strikingly, these early signs also predict poor recovery (53). Our scatter plots of the tactile probing of the hind paws in Supplementary Figure S3 indicate that we have responders and non-responders. Distal pain was observed in approximately half the animals in a study examining the effect of a single disc injury in mice on behavioral measures of pain. Interestingly, increased disc innervation was observed in the responders compared to the non-responders (96). These observations are consistent with observations in humans where IVD degeneration is not always associated with LBP (11). Confirmation of mechanisms underlying these changes are needed to support this theory but also probe why central sensitization processes develop differently, at different times or not at all in humans with pain.

Some generalized and non-evoked measures of pain/discomfort with potential psychosocial links were identified early. At 3 weeks, some DP-2 rats displayed fewer positive interactions with (including aggression toward) a novel female rat, and some showed other physical indices of stress (i.e., excessive weight loss and porphyrin staining on the forehead/nose). From 6–18 weeks, DP-2 (but not DP-1) rats showed altered behaviors during open field gait testing, including escape behaviors, hesitation before walking, freezing >10 sec, and refusal to walk. Further, at week 18, one DP-2 rat displayed escape behaviors during temperature place preference testing. In two other studies of IVD injury, increased grooming, increased periods of immobilization, and “wet dog shakes” behaviors are present by study end at 21 or 42 days after the injury (Table 2) (15, 88). In humans, psychosocial factors are the strongest predictors of poor long-term outcome (97, 98). Some of these factors such as mood and depressive symptoms are thought to have a more influential impact on outcome during the early than later stages of LBP (99). This might be explained by underlying inflammatory processes. For example, inflammation is involved in the exacerbation and pathophysiology of depression (100–103) and, in turn, depression can enhance systemic inflammation (100, 104). This may partly explain why TNFα was elevated most in DP-2 rats and positively correlated with mechanical sensitivity and negative psychosocial behaviors. We have also previously shown that individuals with poorer recovery after an acute episode of LBP have higher depressive symptoms and a unique pro-inflammatory profile (including elevation of TNFα) during the early-acute phase (8, 63, 66) As inflammation and specific psychological features can fuel each other in a bidirectional manner (105, 106), we suspect that a disturbance to either, as might occur with the onset of LBP, could setup a negative cycle between the two that mediates LBP persistence.

Some of the observed pain behaviors might be linked to the release of bioactive substances from the injured disc(s), locally altered biomechanics (e.g., in adjacent vertebral bodies, IVD, facet joints), or both, after injury that causes mechanical or chemical excitation of nociceptor afferents located within or near the injured disc. Puncture of just the annulus fibrosis is thought to produce pain symptoms mainly by altering the mechanical properties of the disc via nucleus pulposus depressurization (18, 19). Moreover, disc herniation can result in the release of the contents of the nucleus pulposus (18, 19) that then sensitize nerve tissue to produce pain symptoms (18, 74). Yet, there is evidence that the herniated disc fragment weight measured intraoperatively in humans does not correlate with the duration of symptoms or severity of pre-operative symptoms (back and leg pain), or post-operative improvements in back pain (107). This further corroborates with our hypothesis that some pain behaviors (e.g., remote sensitivity) are likely explained by central sensitization processes that we have yet to examine in these rats (94).

Serum assays after euthanasia at 18 weeks revealed elevated levels of BDNF and TNFα in DP-2 relative to Sham DP rats. TNFα is a key pro-inflammatory cytokine shown to increase in dorsal root ganglia near disc injury sites between 1 day and 2 weeks after disc puncture (108, 109). TNFα and other proinflammatory cytokines are secreted by cells in degenerating IVDs (37) and contribute to associated pain symptoms (24, 51). Previous studies have investigated the effects of TNFα and its inhibitor, showing that TNFα exacerbates pain and IVD degeneration, while anti-TNF*α* treatments have demonstrated pain relief (110). Additionally, treatment with the anti-TNFα therapeutic, infliximab, attenuates spontaneous signs of focal pain behavior (e.g., reduced locomotion, wet dog shakes, rotation of the animal's head towards the operated leg) after puncture of the L4/5 IVDs in rats (41, 51), and reduces post-injury histological damage (40). Administration of TNFα to cultured DRGs inhibits neurite outgrowth from these DRG, which suggests that TNFα has neurotoxic side effects that may further affect outcomes (111). Although no studies have examined systemic levels of TNFα in animal models of IVD injury, levels are elevated early and remain high in individuals who do not recover after an acute episode of non-specific LBP (8, 63). Interestingly, infliximab treatment via intraperitoneal injection (systemic) also reduces BDNF levels in dorsal root ganglia and the spinal cord after exposing the tissues to herniated nucleus pulposus (112). BDNF is expressed in low levels by nucleus pulposus and annulus fibrosis cells normally, but increases as the disc undergoes degenerative changes or is damaged/injured (42–44, 59). These increases may be reflected systemically as BDNF can “spillover” into circulation and its release is triggered by inflammatory factors such as TNFα (42, 43, 46, 50), which is persistently systemically elevated in individuals who do not recover after an acute episode of LBP (8, 63, 66). This is important because BDNF can sensitize and alter pain pathways at every level via its strong capacity to regulate synaptic plasticity within the peripheral and central nervous system (113). Inappropriate or elevated BDNF expression that persists has been proposed to contribute to maladaptive plasticity processes involved in central sensitization (57)—a required step in the development and maintenance of chronic pain (50, 114). Indeed, higher systemic levels of BDNF correlate with higher generalized sensitivity in animals (115) and in humans experiencing pain (56). These and our findings that BDNF and TNFα were elevated with IVD damage and correlated with remote sensitivity support the hypothesis that sustained elevated levels of circulating BDNF might be a mechanism by which inflammation initiates and maintains central sensitization leading to chronic pain (50). A next step is to examine inflammatory and BDNF profiles in central nervous system tissues from these rats to confirm this hypothesis.

This study has some limitations. First, only female rats were included. As sex is an important factor in the modulation of pain, and sex differences in pain behaviors have been reported in other disc puncture studies (21, 116), sex differences should be considered in future animal studies. This limitation is somewhat mitigated by findings that women bear the burden of chronic LBP (1, 2), sex has been found to inconsistently predict outcomes over 12 months in a human study (117), and no sex differences in disc height and histopathology changes have been reported (21). Our design also makes our findings directly relatable to those of Lillyman et al. (17) who studied the effects of disc injury in female rats across 18 weeks. Second, we assayed for biomarkers in blood collected only at euthanasia at 18 weeks post-injury. This means that we were unable to detect any changes in BDNF and TNFα earlier during the acute and subacute phases in response to IVD injury. Third, we did not investigate nervous system tissues to confirm the presence of peripheral and central sensitization. An investigation of the peripheral and central nervous systems are the next steps to confirm the role of TNFα, BDNF and other mediators of neuroplasticity in the development of pain and chronicity following IVD injury.

## 5 Conclusion

To our knowledge, this is one of the most extensive examinations of pain-related behaviors in a rat model of injury-induced discogenic LBP over a timeframe (18 weeks) that accounts for the acute to chronic transition. Multi-level IVD injury resulted in earlier, prolonged and more pronounced pain behaviors suggestive of more advanced peripheral and central sensitization, than single level IVD injury. BDNF and TNFα may have a role in these relationships but will need to be examined longitudinally and within central nervous system tissues to confirm.

## Data Availability

The raw data supporting the conclusions of this article will be made available by the authors, further inquiries can be directed to the corresponding author/s.

## Supplementary material

Supplementary Figure S1 Scatter plots of histopathological IVD scores for each scored histological feature.

Supplementary Figure S2 Stride length evaluated by measuring the range of individual stride lengths at each time-point. No significant differences were observed between groups or weeks.
