# Supplementary figures and images for: Characterization of pain-related behaviors in a rat model of acute-to-chronic low back pain: single vs. multi-level disc injury
Source: Front Pain Res (Lausanne). 2024 May 6;5:1394017. doi: 10.3389/fpain.2024.1394017 (PMC11102983; doi:10.3389/fpain.2024.1394017)

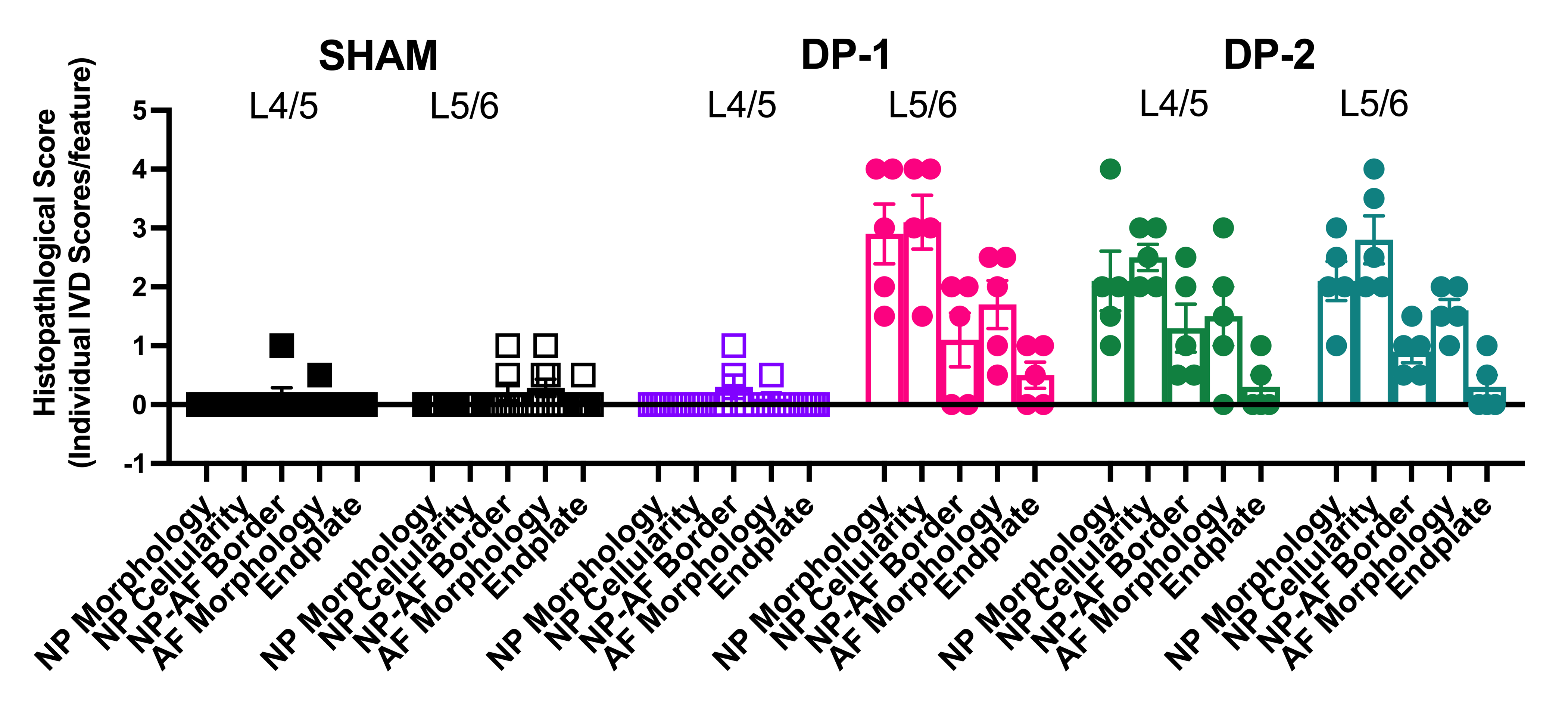

Supplement: Supplementary Figure S1 — Scatter plots of histopathological IVD scores for each scored histological feature. [file Image1.tif]

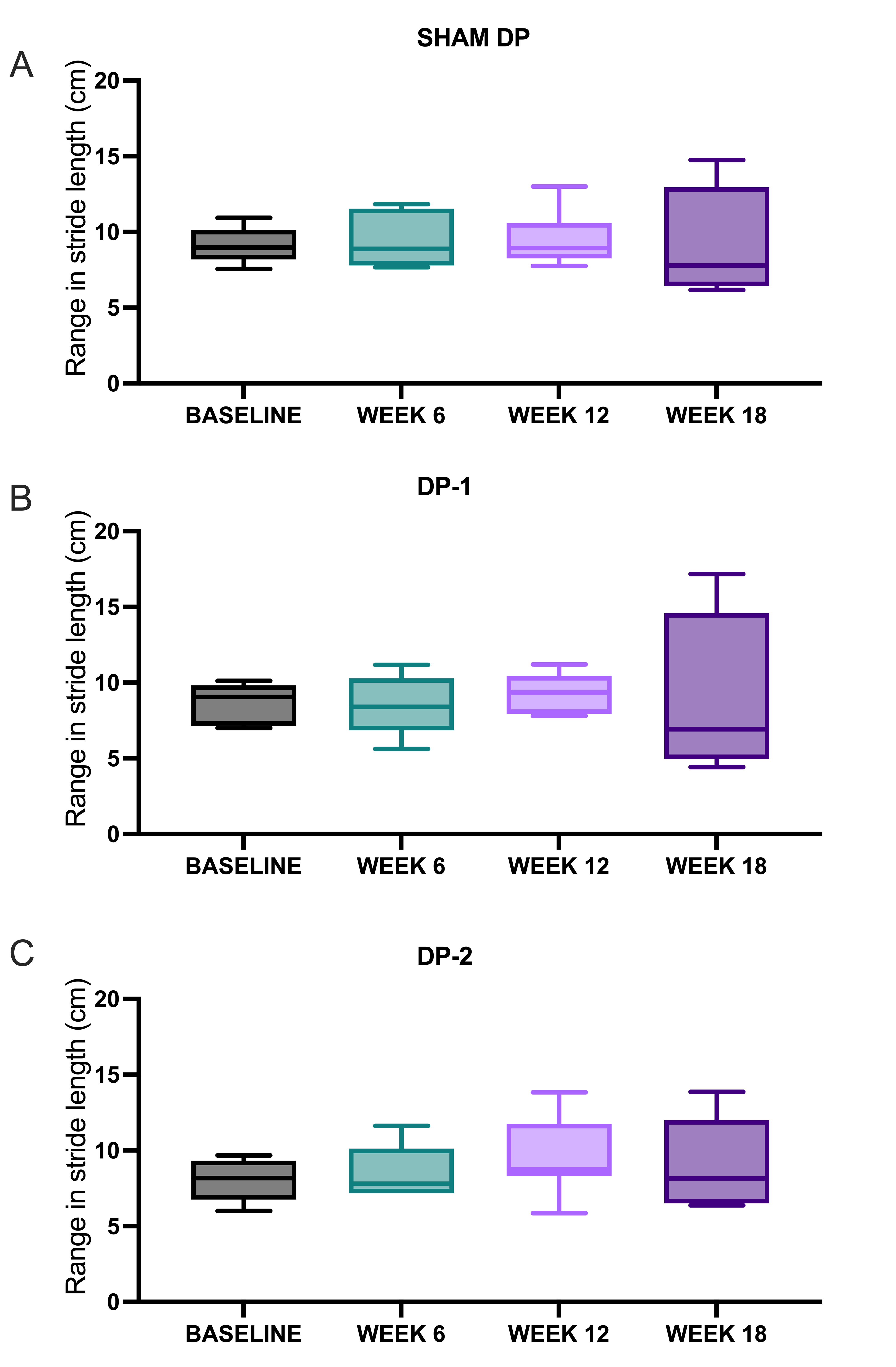

Supplement: Supplementary Figure S2 — Stride length evaluated by measuring the range of individual stride lengths at each time-point. No significant differences were observed between groups or weeks. [file Image2.tif]

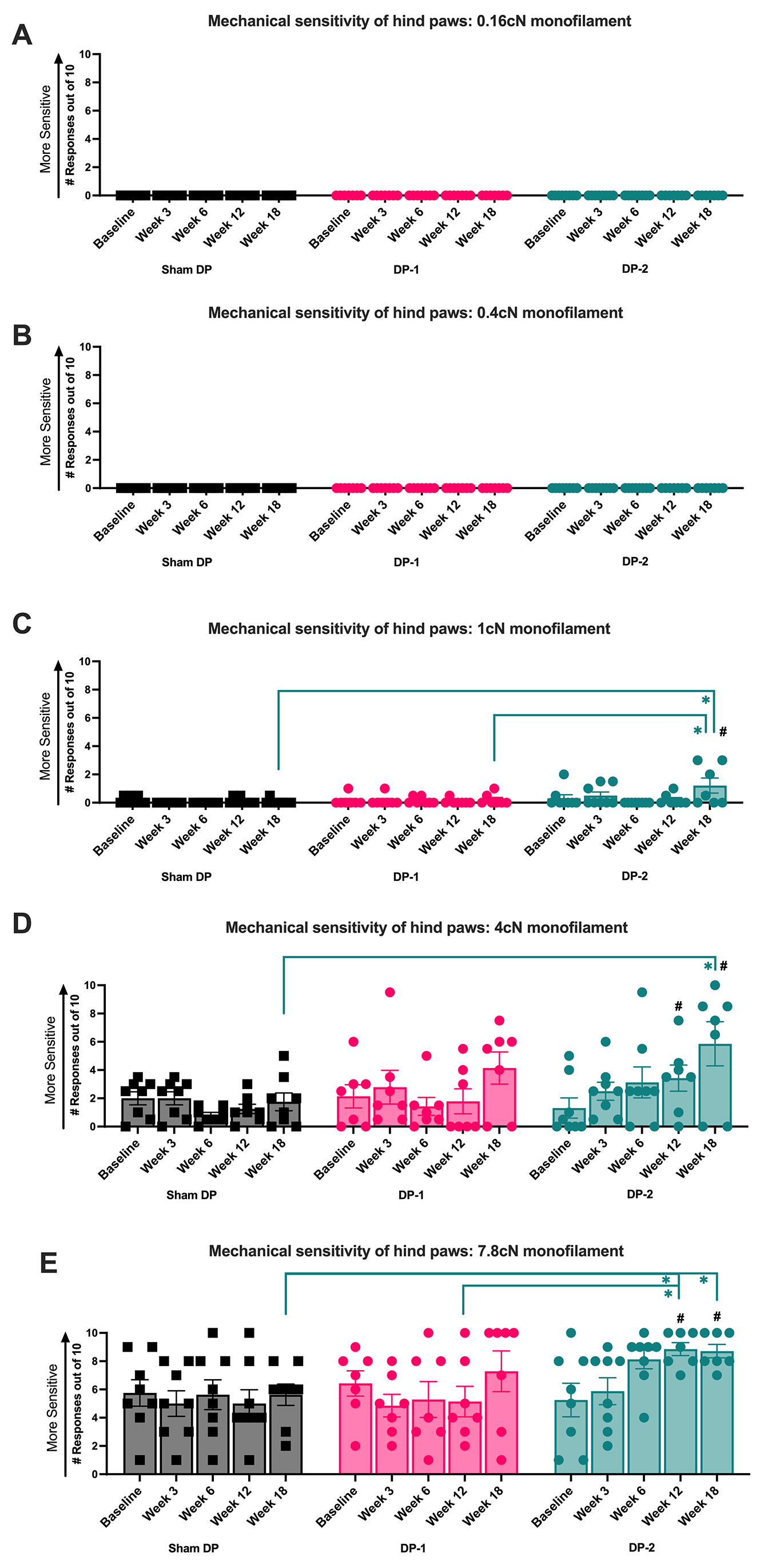

Supplement: Supplementary file 3 [file Image3.tif]
